# Bulk development and stringent selection of microsatellite markers in the western flower thrips *Frankliniella occidentalis*

**DOI:** 10.1038/srep26512

**Published:** 2016-05-20

**Authors:** Li-Jun Cao, Ze-Min Li, Ze-Hua Wang, Liang Zhu, Ya-Jun Gong, Min Chen, Shu-Jun Wei

**Affiliations:** 1 Institute of Plant and Environmental Protection, Beijing Academy of Agriculture and Forestry Sciences, Beijing 100097, China; 2 Beijing Key Laboratory for Forest Pest Control, College of Forestry, Beijing Forestry University, Beijing 100083, China

## Abstract

Recent improvements in next-generation sequencing technologies have enabled investigation of microsatellites on a genome-wide scale. Faced with a huge amount of candidates, the use of appropriate marker selection criteria is crucial. Here, we used the western flower thrips *Frankliniella occidentalis* for an empirical microsatellite survey and validation; 132,251 candidate microsatellites were identified, 92,102 of which were perfect. Dinucleotides were the most abundant category, while (AG)n was the most abundant motif. Sixty primer pairs were designed and validated in two natural populations, of which 30 loci were polymorphic, stable, and repeatable, but not all in Hardy–Weinberg equilibrium (HWE) and linkage equilibrium. Four marker panels were constructed to understand effect of marker selection on population genetic analyses: (i) only accept loci with single nucleotide insertions (SNI); (ii) only accept the most polymorphic loci (MP); (iii) only accept loci that did not deviate from HWE, did not show SNIs, and had unambiguous peaks (SS) and (iv) all developed markers (ALL). Although the MP panel resulted in microsatellites of highest genetic diversity followed by the SNI, the SS performed best in individual assignment. Our study proposes stringent criteria for selection of microsatellites from a large-scale number of genomic candidates for population genetic studies.

Molecular markers are useful tools for population genetics, linkage map construction, gene mapping, and phylogenetics analyses^1^. Microsatellites are widely used among available markers owing to many desirable traits, including a high mutation rate, ease of sample preparation, high applicability to genotyping, and high information content^2^,^3^,^4^. Until recently, the development of microsatellite markers is costly, inefficient, and laborious with conventional methods using microsatellite-enriched libraries^5^,^6^. However, current development in next-generation sequencing technologies have made the genome-scale study of non-model organisms feasible. Whole-genome data accessibility makes the development of novel microsatellite markers faster and less costly, and also makes it possible to survey and characterise microsatellites on a genome-wide scale.

The western flower thrips (WFT) *Frankliniella occidentalis* is an economically important invasive agricultural and horticultural pest. It is native to western North America, but has spread rapidly worldwide through the movement of horticultural material (e.g., cuttings, seedlings, and potted plants) since the late 1970s^7^. Microsatellite markers are commonly used in invasive species to study relations between genetic diversity and invasion success^8^,^9^,^10^,^11^,^12^,^13^, as well as in the reconstruction of invasion histories^14^,^15^. Although nearly 50 microsatellite markers have been developed for WFT^16^,^17^,^18^,^19^, most were developed from expressed sequence tag (EST) sequences. These EST-SSRs (simple sequence repeats) always have few alleles (e.g.^18^,^19^) and are possibly under selection^20^,^21^. This combination generates little information content, and negatively influences the estimation of population genetics parameters. Nonetheless, a large number of loci are necessary to obtain reliable admixture inferences^22^,^23^, which is to be expected in WFT populations because of this species’ high migration frequency^7^. The availability of a draft WFT genome provides opportunities to investigate microsatellites in this species’ genome and to develop potential microsatellite markers with less cost, more efficiency and variability, and greater potential success than with previous methods.

Although the development of microsatellite markers is now more accessible, the criteria for what constitutes good microsatellite markers are still controversial. Empirical studies have shown that selecting the most polymorphic markers, as many researchers often do, will generally overestimate genetic diversity^24^. However, selection strategy choice often has less effect on analyses of population differentiation and clustering^24^. In this study, we aimed to: (i) identify and characterise the microsatellites in the WFT genome; (ii) develop powerful microsatellite markers to examine the genetic structure, genetic diversity, and invasive spread pattern of WFT; and (iii) investigate the selection process for microsatellite markers used in population genetics research.

## Results

### WFT genome microsatellite characteristics

**Figure 1:**
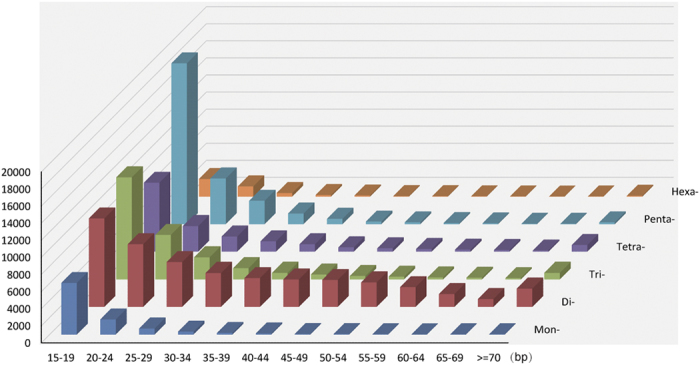
Counts of different motif types with respect to the length of microsatellites.

**Figure 2:**
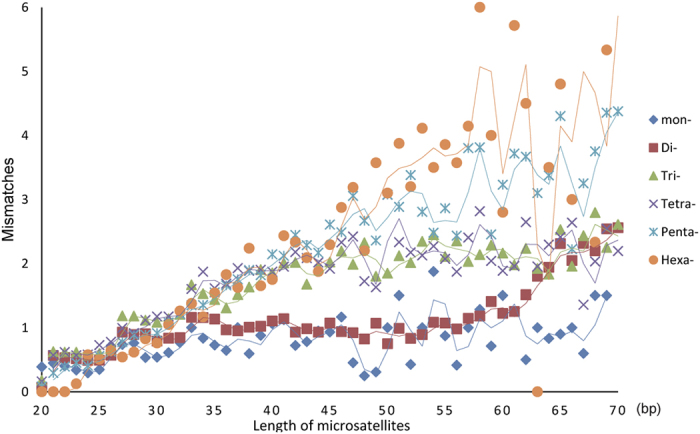
Scatter graph showing the relationship between microsatellite length and motif mismatch.

A total of 132,251 candidate microsatellites were identified from 4,151 WFT genome scaffolds, 92,102 of which were of the perfect type. The total relative abundance was 322.01 loci/Mb. The total relative density was 9.07 Kb/Mb, i.e., ca. 1% of the WFT sampled genome is constructed of microsatellites. Dinucleotides were the most abundant category, having highest density, followed by penta-nucleotides and tri-nucleotides (Table S1). The microsatellite counts decreased as the length of the microsatellites increased, while the rate of decrease of dinucleotides was relatively slower than so of other categories (Fig. 1). The average microsatellite length was 28.18 bp, while most microsatellites (95.1%) were shorter than 61 bp. The relationship between the length of the microsatellite and the percent of imperfect microsatellites or mismatches was next examined. Microsatellites between 20 bp and 70 bp long were used to analyse perfect versus imperfect type, as microsatellites with lengths <20 bp are always perfect under our search parameters. The results show that mismatches continuously increase as length increases for tri-, tetra-, penta-, and hexa-nucleotide motifs (Fig. 2). However, the number of mismatches in mono- and dinucleotide motifs plateaus out after 27 bp and 33 bp, respectively (the dinucleotide mismatch number increases again after 60 bp). A scatter graph of microsatellite lengths versus the percentage of imperfect microsatellites displays the data, illustrating similar interpretations (Figure S1). Mismatches and the percentage of imperfect microsatellites seem to also be associated with the type of motif. The 16 most abundant microsatellite motifs were (AG)n, (AC)n, (CCCCG)n, (AGC)n, (C)n, (CCG)n, (ACAG)n, (AGG)n, (A)n, (ACGCC)n, (ACC)n, (AGGG)n, (CCCGG)n, (AAC)n, (AGGGG)n, and (ACG)n, accounting for 77.5% of all identified microsatellites (Figure S2).

### Development of microsatellite loci

The initial QDD3 program output provided 5,279 primer pairs, of which 4,175 were tri-nucleotide microsatellites and 1,104 were tetra-nucleotide. Following our stringent filter, a total of 381 primers (Appendix S1) flanking perfect microsatellites were retained. We found 305 primer pairs flanking tri-nucleotide microsatellites, and 76 pairs flanking tetra-nucleotide microsatellites. We selected and synthesised 60 primer pairs from different scaffolds for amplification (Appendix S1). In our initial test, 30 primers generated polymorphic genotypes, 23 primers failed to generate amplification in any individual, and seven primers failed in more than two individuals (Appendix S1).

The 30 positive primer pairs were characterised into two WFT populations (Table 1). Each polymorphic locus had four to 17 alleles, with an average of 9.43. The observed heterozygosities (*H*_*O*_) and expected heterozygosities (*H*_*E*_) ranged from 0.1 to 0.875 and 0.348 to 0.895, respectively (Table S2). Significant deviation (after sequential Bonferroni correction^25^, P < 0.05) from HWE was detected in 15 of 60 locus/population combinations, while two locus (WFT4-S31 and WFT4-S57) significantly deviated in both populations (Table S2). In addition, six pairs of loci in 435 pairs (S08 & S20, S16 & S32, S20 & S32, S16 & S36, S33 & S52, S16 & S57) from the MT population and one pair (S03 & S22) from the YQ population showed significant linkage disequilibrium (LD) (after sequential Bonferroni correction, P < 0.05), while none of the locus pairs were significant considering both populations together. In the LOSITAN analyses, one locus (WFT3-S60) fell into the candidate category for balancing selection, and the remaining loci were grouped into the neutral category (Figure S3).

### Performance of four microsatellite panels

Eight loci showed single nucleotide insertions into their alleles (SNI, Table 1). These loci formed the SNI microsatellite panel, while the remaining loci were ranked according to their allele numbers to select the eight most polymorphic markers (MP). Fourteen loci were successfully amplified in all individuals, of which nine did not show SNI. Eight of those nine loci met the criteria for our most stringent strategy (SS) microsatellite panel, except locus WFT3-S01, whose alleles produced ambiguous peaks in GENEMAPPER. We investigated the genetic diversity and structure of two WFT populations using these three panels plus the ALL panel of all developed markers.

**Figure 3:**
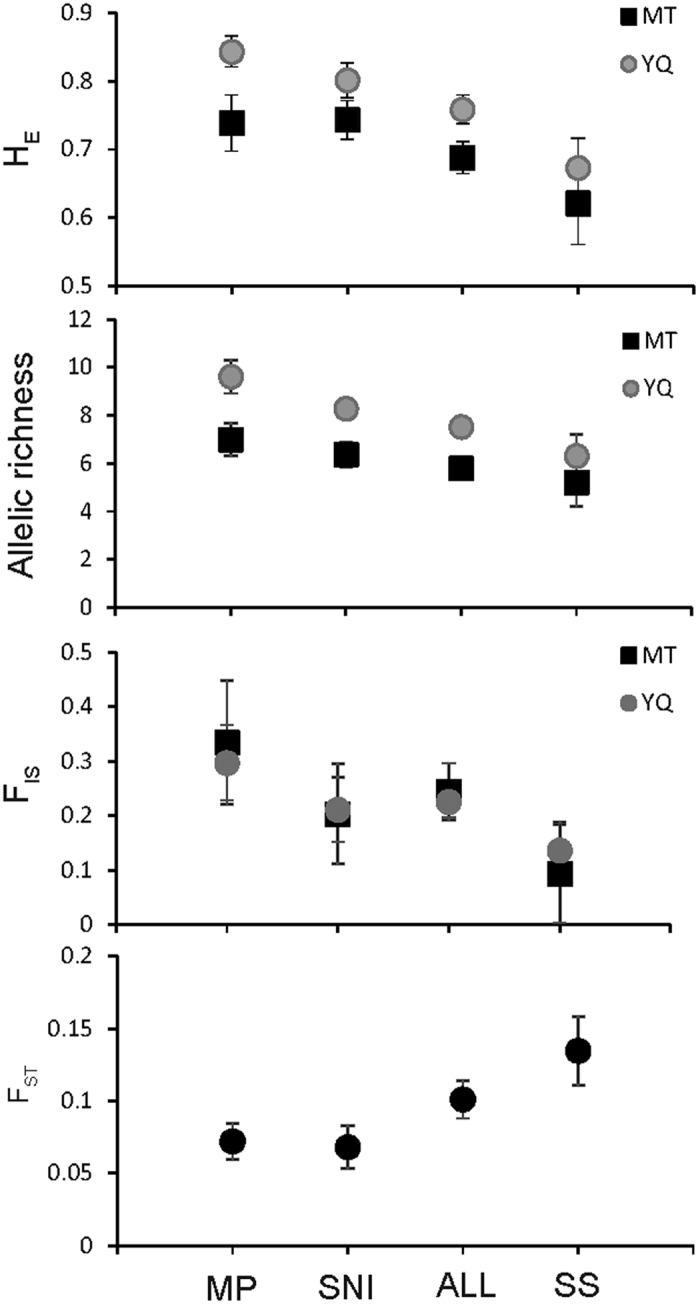
Population genetics parameters inferred for MT and YQ populations using four panels.

The allelic richness (*A*_*R*_) and expected heterozygosity (*H*_*E*_) values were higher for the MT population than for the YQ population in all cases, while both populations have quite similar *F*_*IS*_ values (Fig. 3). Comparison of the microsatellite panels for *A*_*R*_ and *H*_*E*_ in the two populations reveals a decreasing trend in the order of MP, SNI, ALL, and SS values. The MP panel yielded significantly higher *A*_*R*_ and *H*_*E*_ values than the SS and ALL panels in the YQ population (MP vs. SS: *A*_*R*_, W = 53, P = 0.027; *H*_*E*_, W = 56, P =  0.012; MP vs. ALL: *A*_*R*_, W = 182.5, P = 0.025; *H*_*E*_, W = 176, P = 0.045). A significant difference was also observed in SNI versus SS in the YQ population (H_E_, W = 8, P = 0.012). No significant differences were found between the remaining panel pairs in the YQ populations nor in all the MT population panel pairs.

**Figure 4:**
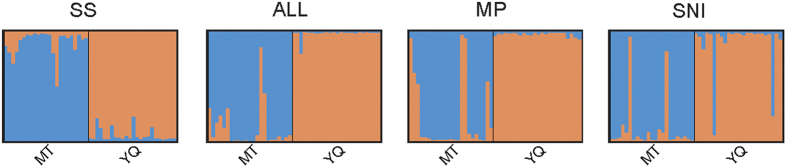
Results of STRUCTURE analyses for MT and YQ populations using four marker panels.

Population differentiation (*F*_*ST*_) showed an increasing trend in order of MP, SNI, ALL, and SS values. However, the differences were not statistically significant. In the STRUCTURE analyses, all panels obtained their highest Delta *K* at *K*  =  2, which suggests two distinct clusters. The average posterior probability (Q) was high in all cases (MT: SS, Q = 0.903; ALL, Q = 0.880; MP, Q = 0.779; SNI, Q = 0.885; YQ: SS, Q = 0.942; ALL, Q = 0.984; MP, Q = 0.979; SNI, Q = 0.899). However, some individuals were not correctly assigned to their respective population. There were 1, 2, 4, and 4 individuals incorrectly assigned for the SS, ALL, MP, and SNI microsatellite markers, respectively (Fig. 4). In the principal component analyses, the ALL and SS panels performed better than the MP and SNI panels in assignment of individuals (Figure S4).

## Discussion

### Genome characteristics

The proportional coverage of different motif types in genomes varies among species^26^. Dinucleotide motifs are the most abundant category in most cases, while the second most abundant are varied among species^26^. Mono-, tri-, and tetra-nucleotide microsatellites have higher coverage than penta-nucleotides in most species^26^, while the penta-nucleotide category is the second most abundant in our study. Furthermore, we found the CCCCG repeat to be the third most abundant microsatellite motif following two dinucleotides (AG, AC) in WFT. The AG repeat is the most frequent microsatellite motif in WFT, while the AC motif is the most frequent in most of other species^26^,^27^.

Mismatches are important in the evolution of microsatellites^28^. Point mutations within the repeat region that cause mismatches will divide the original repeat into shorter ones, which increase locus stability and accelerate the degeneration of long microsatellites^27^,^28^. In this study, mismatches showed a positive correlation with the length of the microsatellite in tri-, tetra-, penta-, and hexa-nucleotide motifs (Fig. 2), as previously reported^29^, although other factors might contribute to the mutation rate between loci^29^. However, mismatches of mono- and dinucleotide microsatellites with lengths between 30 bp and 60 bp were relatively constant. Additionally, the abundance of dinucleotide microsatellites was also relatively constant from 30 bp to 60 bp, which also had a lower percentage of imperfect microsatellites than other types (Fig. 1 and Figure S1). These long, perfect dinucleotide repeats may be less prone to point mutation than other types, or they may be later acquisitions, still in the expansion stage of their life cycle, and potentially close to confronting interruptions and degeneration^30^. The mechanism creating these patterns is outside the scope of this study.

### Criteria for microsatellite selection

Selecting a set of appropriate markers is first step in many molecular ecology studies. Recent improvements in next-generation sequencing technologies, as well as the ready availability of genomic data, have made it possible to select an appropriate marker panel from a huge set of candidates. However, criteria of marker selection are often not mentioned in these studies, or the focus is on the polymorphism of markers^24^. Allelic variation is only one characteristic of microsatellite loci, and may insufficiently represent the performance of a locus under genetic study. Queiros *et al*. suggested that picking the appropriate microsatellite set should primarily take into account the ecological and evolutionary questions studied and selecting the most polymorphic markers will generally overestimate genetic diversity 24. In this study, we compared four marker panel selection criteria for population genetics studies.

The accuracy and stability of genotyping should first be considered prior to determining population genetics parameters. A number of genotyping problems, such as stuttering, split peaks, and low heterozygote peak height ratios^4^, always lead to high error rates and/or excessive manual correction. Tri- and tetra-nucleotide loci were selected for primer design in this study, because stuttering is less of a problem with them than so with dinucleotide loci. However, two loci (WFT3-S01 and WFT3-S21) showed similarly confusing genotyping profiles, and almost all of the individuals required manual correction. Both loci contain the same motif, (AAT)n, which corroborates with another report regarding genotyping profile problems associated with high A+T content^31^. This is probable the cause of deviations from HWE in these loci.

Only perfect loci were considered for primer design in our study. Eight of these loci exhibited an SNI. SNI mutation patterns can be complex, involving insertion/deletion (indel) events in flanking regions or may result from an excess of base substitutions^32^. Although these alleles can be identified manually, it is laborious and inaccurate.

Amplification success rates were considered as an important criterion in our study. Failed amplifications may relate to null alleles (Table S2), which can lower assignment power and affect *F*_*ST*_ accuracy^33^,^34^. Furthermore, complete data are expected to be more informative than deficient.

In our empirical study, the most polymorphic markers (the MP panel) resulted in higher *H*_*E*_ and *A*_*R*_ than the stringently selected markers (the SS panel), while the SNI panel obtained higher polymorphism levels. However, similar *F*_*ST*_ values were estimated by different marker panels, indicating that the approach of microsatellite selection will not influence the analysis of population differentiation, as previously reported^24^. However, the power of assignment was not enhanced in the most polymorphic markers; exactly the opposite was found. The SS panel performed better than the MP and SNI panel in both assignment tests, and even somewhat better than the ALL panel in Bayesian assignment. The low performance in assignment might be caused by high mutation rate in markers from MP and SNI panels, which either leading to genotyping error or deviation of data from the mutation models implemented in the analytical methods.

## Conclusions

We examined and developed a large number of microsatellite markers from the WFT at the genome-wide scale. Our analyses revealed several distinct characteristics regarding the distribution of microsatellites in the species. High-quality primers can feasibly be designed and selected as appropriate markers for further research by taking advantage of the abundant microsatellites that can be isolated from genome sequences. We found that stringent criteria considering amplification efficiency, SNI within loci, and population genetics parameters can obviously improve individual assignment analysis, through a comparison of the performance of different marker panels. Our study provides an example for the bulk discovery of microsatellites and the development of stringent marker selection criteria for population genetics studies.

## Methods

### Sample collection and DNA extraction

A total of 55 adult females of WFT from eight sampled sites were used in the study. Among the specimens, one individual from each of the eight collection sites was used for an initial examination of selected primer pairs, while 47 from two of the collection sites (23 individuals from Mentougou [MT; 116.102 °E, 39.940 °N] and 24 individuals from Yanqing [YQ; 115.975 °E, 40.457 °N]) near Beijing, China, were used for a population-level survey. All samples were stored in 98% alcohol, and frozen at −80 °C. Total genomic DNA was extracted from a single female adult using a DNeasy Blood and Tissue Kit (Qiagen, Germany).

### Identification of microsatellites

We used the 4,151 *F. occidentalis* genome scaffolds (410,700,254 bp) submitted to GenBank by “the *Frankliniella occidentalis* whole genome shotgun (WGS) project” (accession JMDY01000000, http://www.ncbi.nlm.nih.gov/Traces/wgs/?val =  JMDY01#contigs) for microsatellite discovery. Microsatellites were scanned for and identified using the program SciRoKo, with the default fixed penalty motif criteria^35^. Default parameters included a minimum score of 15 and a mismatch penalty of 5, which identified microsatellites of mono-, di-, tri-, tetra-, penta- and hexa-nucleotide motifs. We also considered possible circular permutation motifs and/or reverse complement motifs as same type^36^,^37^. The length of motifs, the length of the microsatellite, the number of mismatches, the relative abundance of the microsatellite (the SSR number per Mb), and the relative density of the microsatellite (the SSR length [in bp] per Mb) were analysed, to characterise microsatellites in the WFT genome.

### Primer design

The QDD3 program^38^ was used to extract microsatellites along with their flanking sequences (300 bp each) from the scaffolds, and to design the primers.

Genotyping accuracy and stability are associated with target region motifs. Dinucleotide microsatellites are prone to polymerase slippage during the amplification process, which may lead to mistyping^39^,^40^. This is particularly severe when the quality or concentration of the DNA sample is low^41^,^42^, as it often occurs with small insects. Tri- and tetra-nucleotide loci perform better than dinucleotides in this respect^43^,^44^. Our primer design criteria are as follows: (i) only tri- and tetra-nucleotide motifs were considered for primer design; (ii) the repeat number needed to be larger than seven; (iii) the primer needed to be between 18 bp and 24 bp long with an optimal length of 20 bp; (iv) the primer annealing temperature had to be between 58 °C and 62 °C with an optimal of 60 °C; and (v) the annealing temperature difference between pairwise primers had to be <4 °C. All other parameters were used at their default settings.

Imperfect (those with repeat region interruptions), compound, and multiple microsatellites within an amplified region may generate complex genotypes. Therefore, output primers were further filtered manually using the following, more stringent, criteria: (i) only perfect (without repeat region interruptions) loci were retained; (ii) the minimum distance between the 3′ end of two primer pairs and their target region had to be >10 bp; and (iii) primers using the ‘A’ design strategy that do not have multiple microsatellites, nanosatellites, or homopolymers in the amplicon were retained.

### Polymorphic microsatellite isolation

Sixty primer pairs were selected and synthesised for validation. A universal primer (CAGGACCAGGCTACCGTG) was added to the 5′ end of the forward primers following the method of Blacket *et al*.^45^.

We initially tested for primer effectiveness and polymorphism level using one individual from each of the eight different sampling sites. The amplifications were performed using the GoTaq Green Master Mix (Promega, USA) in a final volume of 10 μl. The polymerase chain reaction (PCR) reaction mixture contained three primers: 0.08 μl forward primer, 0.16 μl reverse primer, and 0.32 μl universal primer labelled with fluorescence (FAM, HEX, and ROX sequencing dyes). Our PCR protocol follows: 5 min at 95 °C, followed by 35 cycles of 95 °C for 30 s, 56 °C for 40 s, and 72 °C for 40 s, with a final extension at 72 °C for 15 min. PCR products were analysed on an ABI 3730xl DNA Analyzer (Applied Biosystems, USA) using the GeneScan 500 LIZ size standard (Applied Biosystems, USA). Genotyping was conducted using GENEMAPPER 4.0 (Applied Biosystems, USA). Primer pairs that failed in more than two individuals, or that were monomorphic in eight individuals, or that produced more than two peaks were discarded. Those remaining primer pairs that generated polymorphic genotypes were further analysed in the next step.

For the next step, 48 female adults from two populations were used to analyse the genetic characteristics of each primer pair. The PCR reaction mixture, cycling conditions, and genotyping methods were identical to those of the previous step. We repeated failed amplifications twice to eliminate occasional technical mistakes.

### Genetic diversity

The genotyping data of two populations were analysed. The macros Microsatellite Tools^46^ was used to calculate the number of alleles, and the observed heterozygosity (*H*_*O*_) and expected heterozygosity (*H*_*E*_). Allelic richness and the inbreeding coefficient of an individual relative to the subpopulation (F_IS_) were obtained using FSTAT 2.9.3^47^. Null allele frequencies for each loci in each population were estimated using FREENA^33^, with 1,000 bootstrap replicates. The Hardy–Weinberg equilibrium (HWE) at each locus/population pair, the linkage disequilibrium (LD) between each pair of loci within each population, and the pairwise mean population differentiation (*F*_*ST*_) were tested and/or calculated using GENEPOP 4.2.1^48^. Putative loci under selection were detected based on the *F*_*ST*_ outlier method employed in the program LOSITAN^49^ with two options: “force mean *F*_*ST*_” and “neutral mean *F*_*ST*._”.

### Population structure

We examined population genetic structure using STRUCTURE 2.3.4^50^. Admixture models with correlated allele frequencies and no prior location information were used for the analyses. The clustering test was replicated 30 times under each *K* value (from 1 to 5) with 200,000 Markov chain Monte Carlo iterations, after a burn-in of 100,000 iterations. The Delta (*K*) method^51^ was used to estimate optimal *K* values by submitting the Structure output to Structure Harvester Web 0.6.94^51^. The membership coefficient matrices (Q-matrices) associated with the optimal *K* values inferred from the previous step were processed using CLUMPP 1.1.2^52^ with its Greedy algorithm, and then visualised with the program DISTRUCT 1.1^53^. A posterior threshold probability (Q-value of individuals obtained with CLUMPP) of 0.60 was used to evaluate whether individuals were correctly assigned to their respective population. A principal component analysis (PCA) test was also performed using the package Adegenet 1.4–2^54^ in R environment^55^, which does not require assumptions on the Hardy–Weinberg and linkage equilibrium.

### Criteria for selection of marker panel

Six factors were taken into account for the selection of our microsatellite markers: HWE, LD, polymorphism, amplification fail rate, single nucleotide insertions (SNI), and peak patterns genotyped in GENEMAPPER. We constructed three microsatellite panels based on three criteria for each locus (Table 1): (i) the most polymorphic markers (MP); (ii) markers showing SNI; and (iii) stringently selected markers (SS), which were those that did not significantly deviate from HWE in all populations, generated amplification in all individuals, did not show SNIs, and had unambiguous GENEMAPPER peaks. Genetic diversity and population structure were estimated using these three microsatellite panels. Finally, the whole set of 30 markers developed in this study (ALL) were analysed. A comparison of the effect of marker selection was performed using the nonparametric Mann–Whitney–Wilcoxon test in the R environment.

## Additional Information

**How to cite this article**: Cao, L.-J. *et al*. Bulk development and stringent selection of microsatellite markers in the western flower thrips *Frankliniella occidentalis. Sci. Rep.* **6**, 26512; doi: 10.1038/srep26512 (2016).

## Supplementary Material

Supplementary Information

## Figures and Tables

**Table 1:** Thirty microsatellite loci developed for west flower thrips.

**Locus**	**Scaffold**	**Motif**	**Forward primer 5′** = **>3′**	**Reverse primer 5′** = **>3′**	**Size (bp)**	**N**_**A**_	**Tm (°C)**	**N**	**FL**	**Panel**
wft3-S01^#^	KL023705	(AAT)12	TCAATGAGTAGTGGCAGTCGG	GTAGCTAGTGAGGCAGGCTG	162–246	16	56	47	HEX	MP
wft3-S03	KL023701	(AGC)12	CCGACAGGAACACGTGGTCT	AATCGCAAACAGAATCCGACG	135–188	8	56	45	HEX	
wft3-S06	KL023747	(AGC)8	TCCGATGACGCCAACTTACC	CACTCGTCCTCGGCTTCAG	142–159	5	56	46	HEX	
wft3-S08	KL023707	(ACG)8	GAAGCTGCTGTGACTCCAGT	GACGCAGAGAACGACCCTG	155–173	5	56	47	HEX	SS
wft4-S09^#^	KL023695	(AGGC)7	GGCCGATGATTGTGCAAACA	CCGCATGCTAGCAATCCACT	165–185	5	56	47	HEX	SS
wft4-S13	KL023711	(AACC)16	TGTGCGGTTTCATGCAAAGG	CGAGACAAACGGGTGGATGA	157–184	4	56	47	HEX	SS
wft3-S14	KL023717	(AAG)22	TTCTCGCTCTTCAGGCGAAA	AGTATGGATTTGGCGGCGTT	178–222	10	56	47	HEX	SS
wft4-S16	KL023774	(ACAG)14	CAAGCCACTCCCAGGAGATG	GACAGACGACATGACCTCGG	190–223	8	56	43	HEX	
wft4-S17	KL023720	(ACAG)20	GACCGTCAACGTGGACCC	CCGACTGGACTGCTACTGAC	99–249	10	56	46	HEX	
wft3-S20	KL023696	(AGC)17	AGCGCATTGTCCAGGCTAAT	GCAGCGTGTTTGCAGTATGT	204–271	6	56	46	HEX	
wft3-S21^#^	KL023772	(AAT)15	GGTGACGTTGAACAAACCGA	GAGGAGCCAACCCAATGTGA	243–284	11	56	45	FAM	MP
wft4-S22^#^	KL023713	(AGAT)9	CGTTACCGATGTGCCACGTA	ACCTAGTGGATCCCTCGAAAGA	240**–**300	13	56	46	FAM	SNI
wft4-S26^#^	KL023743	(ACAG)17	TTAACGGCGGTCATGCTTCT	AATGCGGCGCTTCGTTAGAA	220–321	11	56	44	FAM	MP
wft3-S27^#^	KL023766	(ACC)13	GGAAGACCAATCATCGCGGA	ATTCGTGCTGCAGTTGGAGT	224–263	10	56	44	FAM	SNI
wft3-S28	KL023710	(ACT)10	TCCACTTGGCGTCAAAGTGT	CAGGCCTGTTTCTGGTCGG	236–253	10	56	47	FAM	SNI
wft4-S29	KL023712	(ACGC)11	CATCACGACAACAATGCCGG	AGCGTCATTATACCGGTGCC	233–272	9	56	41	FAM	
wft4-S30^#^	KL023745	(ACGG)11	TGTAGTAGGCGGGAAATGATGA	GAGTGTCGCAGCAGAACTCT	217–336	11	56	47	FAM	SNI
wft4-S31^##^	KL023716	(ACTC)13	ATCACTTCGCTAGCACGCTC	AGTTACGTCGTTCCGTGTCC	209–261	11	56	41	FAM	MP
wft4-S32^#^	KL023729	(ACAG)16	GTCTCGGTATGCGTACAGGC	ATTTCGATACCAGGCCGTGT	212–359	17	56	45	FAM	MP
wft3-S33	KL023736	(AGC)10	TCGGAATAACGCTGAGTGCC	TAGGTGCTCTGCAGATGGAC	238–255	10	56	47	FAM	SNI
wft4-S34	KL023718	(AGAT)13	GCTGCACGCTAAGTTCACAC	GTTGCAGCTCTTCTCACCTG	234–273	9	56	46	ROX	
wft4-S36	KL023722	(AAAG)10	CCGGCAGCACGTTTATCAAA	TTGCGGTTGATTCGTTGCAT	275–293	6	56	46	ROX	
wft3-S43	KL023742	(AGC)20	GAGCACGCCACGATGATGAA	GACGGATGGAAGGACGCAAT	258–299	11	56	47	ROX	MP, SS
wft4-S45	KL023755	(ACAG)8	ACCCAAATACGGCAACCAAC	ATCGGTGCACAATCAGACGG	296–320	6	56	47	ROX	SS
wft4-S50	KL023714	(ATCC)13	CCTTGCACGCTCTGATAGGT	TCCCGTAGTTGGCCAAATGA	303–351	7	56	47	ROX	SS
wft4-S52	KL023739	(AAAC)11	AGGGCGTTGATGTTGAGGAA	CGGCGTGATCTAGAGGGTCT	312–366	10	56	39	ROX	SNI
wft3-S53^#^	KL023752	(AAC)17	ACTCCGTACACAAGATGGAGT	AGTGCGGATCTCAGGCTAAC	299–357	11	56	47	ROX	SNI
wft4-S57^##^	KL023777	(AGAT)23	GACGGAGAGGGATTCGTCAC	GCTGCTCATGCGACAAATGA	338–416	13	56	43	ROX	MP
wft4-S58	KL023694	(AGAT)15	AAGCCGAATGGGAGACACTT	ACACGTGAACAGCGTATAGGT	338–394	12	56	47	ROX	MP, SS
wft3-S60	KL023732	(AGC)8	AGCTCTTGCGGTGATGATCC	AATTGATCGCAGCTGTCAGC	358–383	8	56	47	ROX	SNI

Tm, annealing temperature; NA, number of alleles; N, number of individuals successfully genotyped from the 48 insects; ^#^locus that deviates from Hardy–Weinberg equilibrium significantly in one population; ^##^locus that deviates from Hardy–Weinberg equilibrium significantly in two population (after sequential Bonferroni correction for multiple tests, P < 0.05); Panel, marker panels; FL, fluorescent label.
