# Supplementary material for: Bulk development and stringent selection of microsatellite markers in the western flower thrips Frankliniella occidentalis
Source: Sci Rep. 2016 May 20;6:26512. doi: 10.1038/srep26512 (PMC4873785; doi:10.1038/srep26512)
Supplement: Supplementary Information [file srep26512-s1.pdf]

Bulk development and stringent selection of microsatellite markers in the western flower thrips *Frankliniella occidentalis*

Li-Jun Cao [1, 2], Ze-Min Li [1], Ze-Hua Wang [1], Liang Zhu [1], Ya-Jun Gong [1], Min Chen [2, *], Shu-Jun Wei [1, *]

[1] Institute of Plant and Environmental Protection, Beijing Academy of Agriculture and Forestry Sciences, Beijing 100097, China

[2] Beijing Key Laboratory for Forest Pest Control, College of Forestry, Beijing Forestry University, Beijing 100083, China

Email addresses: Li-Jun Cao: gmatjhpl@163.com

Ze-Min Li: 805508851@qq.com

Ze-Hua Wang: wangzehua200707@163.com

Liang Zhu: zhuliang25@sina.com

Ya-Jun Gong: gongyajun200303@163.com

Min Chen: minch@bjfu.edu.cn

Shu-Jun Wei: shujun268@163.com

Table S1. Distribution of microsatellites in the *Frankliniella occidentalis* genome.

Motif	Mon-	Di-	Tri-	Tetra-	Penta-	Hexa-	Total
Counts	9,449	46,488	24,454	17,597	29,904	4,359	132,251
Average length (bp)	20.58	35.18	25.35	28.07	22.54	24.88	28.18
Abundance(Counts/Mbp)	23.01	113.19	59.54	42.85	72.81	10.61	322.01
Percent of each type	7.14%	35.15%	18.49%	13.31%	22.61%	3.30%	

Table S2. Population genetics parameters for two *Frankliniella occidentalis* populations at 30 loci.

locus	MT						YQ					
	N	N_A	H_O	H_E	F_{IS}	Null	*N*	N_A	H_O	H_E	F_{IS}	Null
wft3-S01	23	9	0.826	0.836	0.012	0.031	24	14	0.583	0.879	0.341	0.148*
wft3-S03	21	6	0.429	0.613	0.306	0.085	24	8	0.583	0.759	0.235	0.097
wft3-S06	22	4	0.500	0.692	0.283	0.103	24	4	0.417	0.566	0.269	0.076
wft3-S08	23	3	0.565	0.476	-0.192	0.000	24	5	0.750	0.738	-0.016	0.000
wft4-S09	23	4	0.348	0.600	0.426	0.148*	24	4	0.500	0.648	0.232	0.082
wft4-S13	23	2	0.174	0.348	0.506	0.144	24	4	0.417	0.632	0.346	0.120
wft3-S14	23	6	0.826	0.740	-0.119	0.000	24	9	0.667	0.728	0.086	0.067
wft4-S16	20	5	0.100	0.585	0.833	0.312*	23	8	0.609	0.776	0.219	0.102
wft4-S17	22	8	0.409	0.775	0.478	0.208*	24	8	0.792	0.863	0.084	0.047
wft3-S20	23	3	0.348	0.371	0.064	0.024	23	6	0.522	0.646	0.196	0.074
wft3-S21	23	5	0.348	0.770	0.554	0.239*	22	10	0.500	0.864	0.427	0.188
wft4-S22	23	10	0.652	0.865	0.250	0.109	23	10	0.435	0.772	0.442	0.194*
wft4-S26	23	7	0.478	0.642	0.259	0.115	21	9	0.500	0.863	0.427	0.188*
wft3-S27	23	6	0.826	0.760	-0.089	0.000	21	8	0.476	0.815	0.422	0.190*
wft3-S28	23	6	0.565	0.599	0.058	0.039	24	9	0.875	0.852	-0.028	0.000
wft4-S29	23	5	0.652	0.698	0.067	0.040	18	6	0.389	0.578	0.333	0.148
wft4-S30	23	7	0.348	0.798	0.570	0.246*	24	9	0.500	0.695	0.285	0.093
wft4-S31	18	7	0.111	0.783	0.862	0.373*	23	7	0.435	0.840	0.488	0.216*
wft4-S32	23	9	0.696	0.771	0.100	0.079	22	12	0.727	0.888	0.185	0.098*
wft3-S33	23	6	0.783	0.680	-0.155	0.000	24	10	0.708	0.887	0.205	0.085
wft4-S34	23	7	0.565	0.731	0.231	0.048	23	7	0.609	0.811	0.253	0.103
wft4-S36	23	5	0.565	0.738	0.238	0.104	23	5	0.739	0.786	0.062	0.002
wft3-S43	23	5	0.435	0.510	0.151	0.060	24	8	0.667	0.692	0.038	0.000
wft4-S45	23	4	0.652	0.666	0.021	0.028	24	6	0.417	0.621	0.333	0.103
wft4-S50	23	7	0.826	0.748	-0.107	0.000	24	5	0.417	0.449	0.073	0.006
wft4-S52	21	6	0.429	0.715	0.407	0.153	18	9	0.722	0.895	0.198	0.088
wft3-S53	23	8	0.435	0.770	0.441	0.184*	24	9	0.708	0.746	0.052	0.040
wft4-S57	21	6	0.238	0.720	0.675	0.277*	22	11	0.455	0.848	0.470	0.210*
wft4-S58	23	11	0.826	0.880	0.063	0.008	24	11	0.875	0.878	0.003	0.000
wft3-S60	23	5	0.652	0.760	0.145	0.071	24	7	0.667	0.751	0.114	0.026

N, the number of insects successfully genotyped from the 24 insects; N_A, number of alleles detected in the population; H_O, observed heterozygosity; H_E, expected heterozygosity; F_{IS}, inbreeding coefficient; Null, null allele frequency; *Loci with significant deviations from Hardy–Weinberg equilibrium after sequential Bonferroni correction for multiple tests ($P < 0.05$).

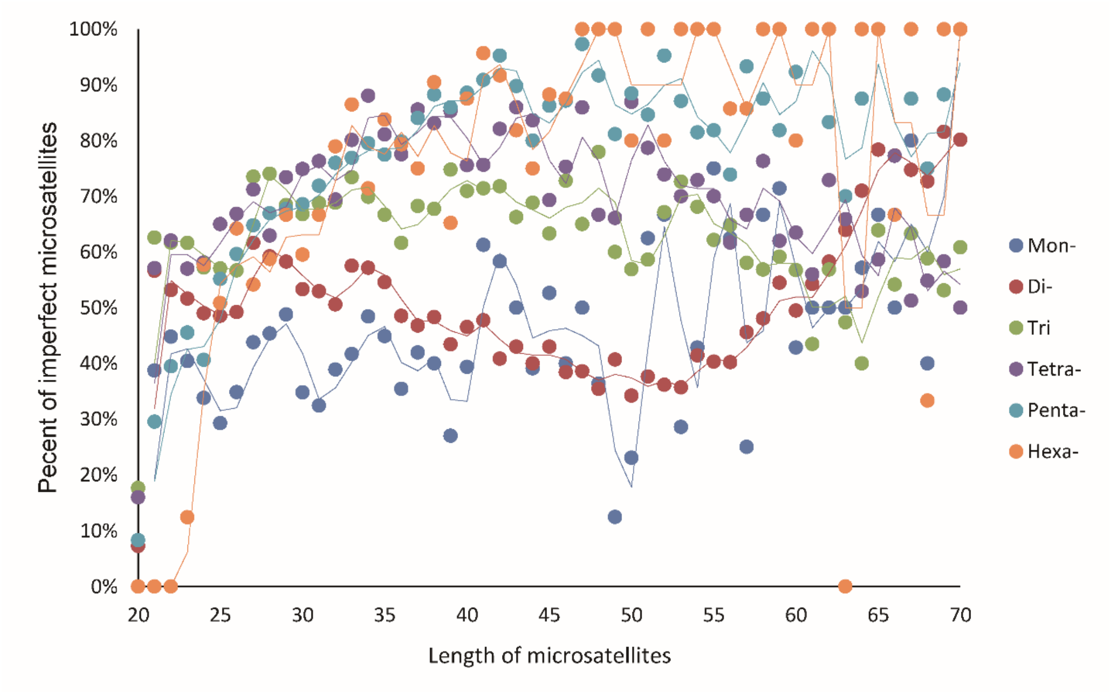

Figure S1 Scatter graph showing the relationship between microsatellite length and percentage of imperfect microsatellites.

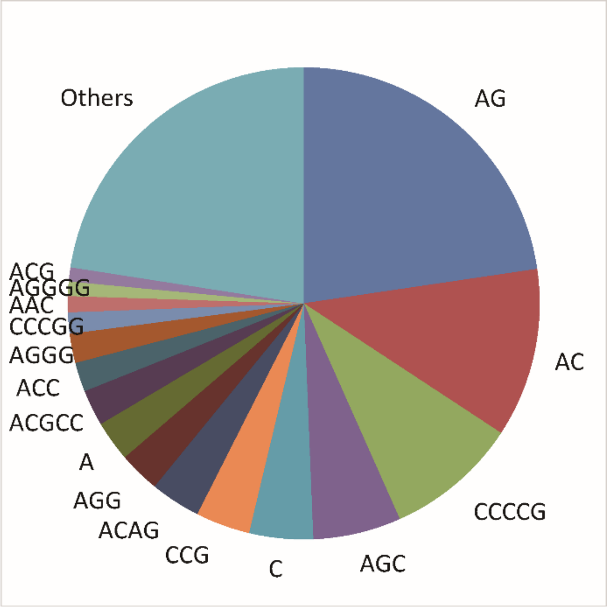

Figure S2 Frequency of microsatellite motif categories in *Frankliniella occidentalis*.

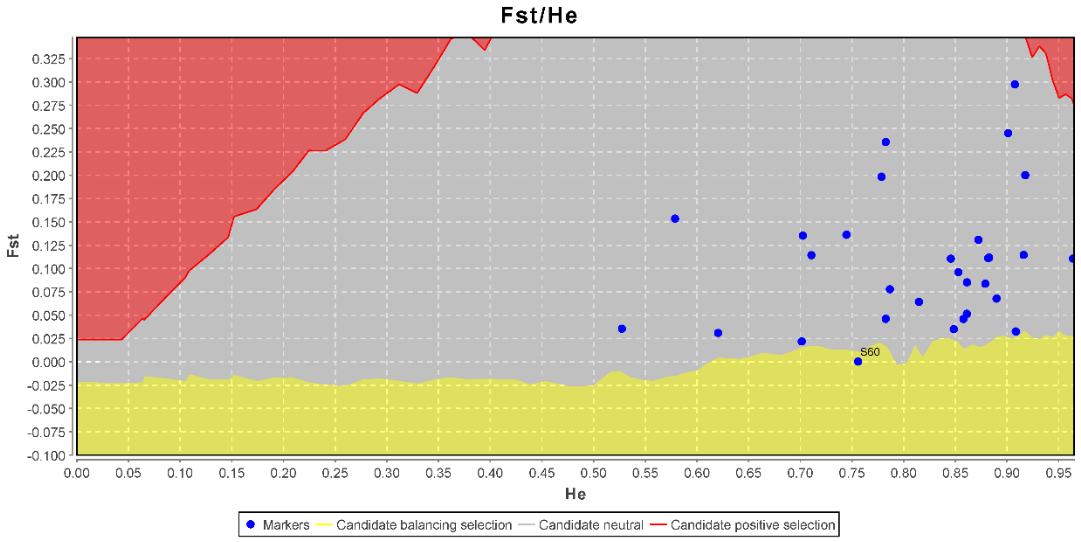

Figure S3 LOSITAN results for 30 loci in two *Frankliniella occidentalis* populations. Loci falling in the red region are candidates for positive selection, yellow for balancing selection, and grey for neutral.

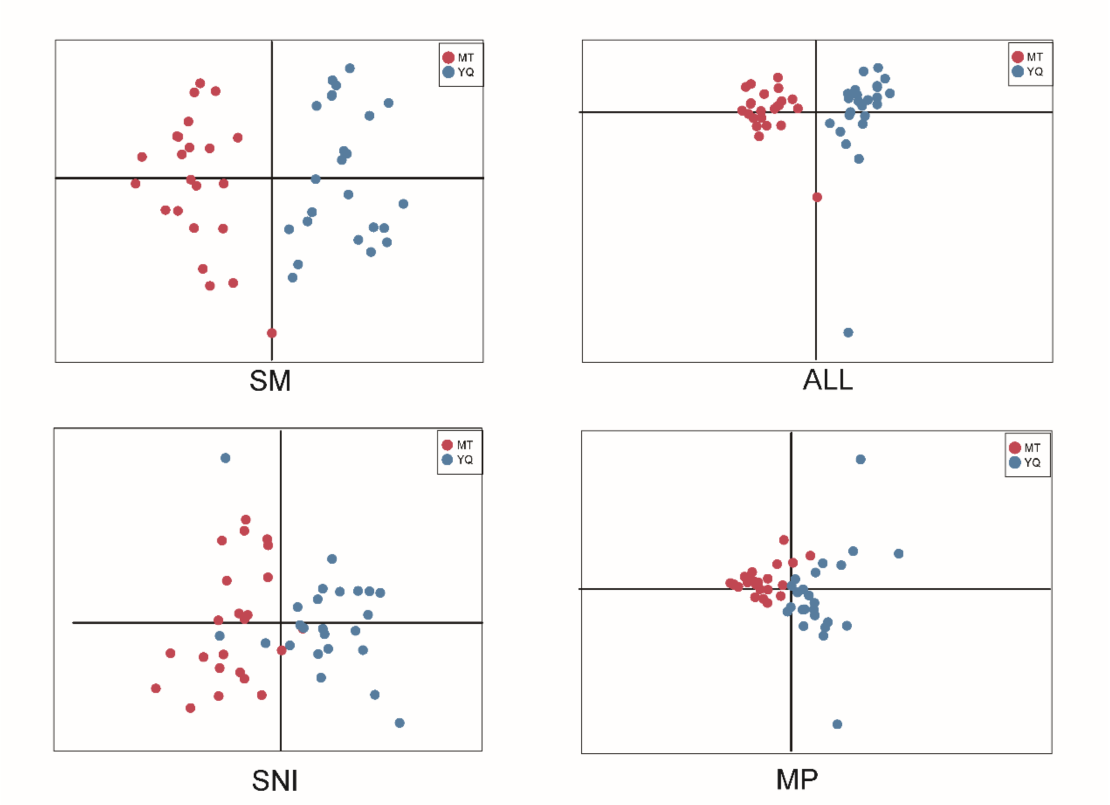

Figure S4 Principal component analysis results for MT and YQ populations of *Frankliniella occidentalis* using four marker panels.

Appendix S1. Primers designed in the study

SEQUENCE_CODE	Locus used in this study	Amplification	MOT_TRANS	PRIMER_LEFT_SEQUENCE	PRIMER_RIGHT_SEQUENCE
gi646098144gbKL023705.1_809287	wft3_QDD_S01	polymorphism	AAT	TCAATGAGTAGTGGCAGTCGG	GTAGCTAGTGAGGCAGGCTG
gi646098085gbKL023764.1_1132448	wft3_QDD_S02	non-amplification	AGC	GTTCTCGTCACCACCAGCTC	TCAGTGGGATTAGCGTGCAG
gi646098148gbKL023701.1_1462119	wft3_QDD_S03	polymorphism	AGC	CCGACAGGAACACGTGGTCT	AATCGCAAACAGAATCCGACG
gi646098123gbKL023726.1_1264175	wft3_QDD_S04	non-amplification	AGG	CGGCACCAGCTCGTCTAG	CGAAGAAGAGGGCGAGGATG
gi646098102gbKL023747.1_1249990	wft3_QDD_S06	polymorphism	AGC	TCCGATGACGCCAACTTACC	CACTCGTCCTCGGCTTCAG
gi646098084gbKL023765.1_854093	wft3_QDD_S07	low-success-rate	AGG	TAAGGAAAGGAATCGGGCGG	GAGGGAGAGGGAGGGTGAT
gi646098142gbKL023707.1_2775638	wft3_QDD_S08	polymorphism	ACG	GAAGCTGCTGTGACTCCAGT	GACGCAGAGAACGACCCTG
gi646098145gbKL023704.1_46455	wft3_QDD_S10	non-amplification	AGC	ACCCGGATGTCGTTTCAGAC	GACAAGAGGCGACTTCCTGG
gi646098114gbKL023735.1_54875	wft3_QDD_S11	non-amplification	ACC	CGGATCTAGGCCGGTGTAGT	TCCTATTACTTGTGGACACCCA
gi646098125gbKL023724.1_2387656	wft3_QDD_S12	low-success-rate	AGG	TCCATCCCATAAATCCTTCGGG	TCCTCGCCACGATTACCTTT
gi646098132gbKL023717.1_382966	wft3_QDD_S14	polymorphism	AAG	TTCTCGCTCTTCAGGCGAAA	AGTATGGATTTGGCGGCGTT
gi646098093gbKL023756.1_704979	wft3_QDD_S18	low-success-rate	AGC	CGGGATGCACCATATCACCA	CCAAGGGCTGCAGGAAGATG
gi646098153gbKL023696.1_895764	wft3_QDD_S20	polymorphism	AGC	AGCGCATTGTCCAGGCTAAT	GCAGCGTGTTTGCAGTATGT
gi646098077gbKL023772.1_324200	wft3_QDD_S21	polymorphism	AAT	GGTGACGTTGAACAAACCGA	GAGGAGCCAACCCAATGTGA
gi646098076gbKL023773.1_1011265	wft3_QDD_S23	non-amplification	AAT	GGGTCACGAATGAAATCATGGT	AGTGCCAATAGTCTTTCCCTGT
gi646098112gbKL023737.1_1048453	wft3_QDD_S24	non-amplification	AGC	CCTGTTTGCGAGGCTGTTTC	GCAGGTGAGTGAGGTGTGAT
gi646098083gbKL023766.1_994338	wft3_QDD_S27	polymorphism	ACC	GGAAGACCAATCATCGCGGA	ATTCGTGCTGCAGTTGGAGT
gi646098139gbKL023710.1_3280802	wft3_QDD_S28	polymorphism	ACT	TCCACTTGGCGTCAAAGTGT	CAGGCCTGTTTCTGGTCGG
gi646098113gbKL023736.1_1043615	wft3_QDD_S33	polymorphism	AGC	TCGGAATAACGCTGAGTGCC	TAGGTGCTCTGCAGATGGAC
gi646098118gbKL023731.1_41056	wft3_QDD_S35	low-success-rate	AAG	CGTGCTGCGGAGATTTATGG	CCACAGCTCTGATTGCCCAA
gi646098126gbKL023723.1_2042073	wft3_QDD_S38	non-amplification	AGG	CCAGTGCAGGAGACATGCAT	CGTAACCGCCATGATAGCCA
gi646098119gbKL023730.1_1414197	wft3_QDD_S39	non-amplification	AAG	GAGTGCGAGTGGGACAGAC	CGATGCGGTGTCGAACAATT
gi646098074gbKL023775.1_8435	wft3_QDD_S40	non-amplification	AGC	AAATCTCTTGGCGCGATGGG	TCACATCTAGCCGTCGCATC
gi646098108gbKL023741.1_1625980	wft3_QDD_S42	non-amplification	AGG	CGTGCCAACAAGTTTACTACGG	GCAAGGCTGCTATTCGAGAAG
gi646098107gbKL023742.1_1197280	wft3_QDD_S43	polymorphism	AGC	GAGCACGCCACGATGATGAA	GACGGATGGAAGGACGCAAT
gi646098141gbKL023708.1_736613	wft3_QDD_S44	non-amplification	CCG	CTCCCTGAAACCAACCGGAA	GACGTCAGTCAGTTTGCGTC
gi646098134gbKL023715.1_372425	wft3_QDD_S46	non-amplification	AGC	CCACGAGACGAGGCGATTAA	CGCAGCGCATACCTGATTTC
gi646098090gbKL023759.1_1330594	wft3_QDD_S47	non-amplification	AGC	CCGCGATATAAACAGGCCCA	CCAATTTCACACGCCTGCTC
gi646098130gbKL023719.1_2851065	wft3_QDD_S48	non-amplification	AGC	CCTCGGCGATAGTGTGGG	CAGGGCGGTTCAGCATAGTT
gi646098097gbKL023752.1_1267754	wft3_QDD_S53	polymorphism	AAC	ACTCCGTACACAAGATGGAGT	AGTGCGGATCTCAGGCTAAC
gi646098124gbKL023725.1_869515	wft3_QDD_S55	non-amplification	AAG	AAGGAGTTTCCGGGTTCCAC	CGCCGGAAATGAACCATCAC
gi646098140gbKL023709.1_1089874	wft3_QDD_S56	non-amplification	ATC	TGCCCAGGTCAATCCGTTAC	TACGTCCTGCTCTTCTACGC

gi646098117gbKL023732.1_788774	wft3_QDD_S60	polymorphism	AGC	AGCTCTTGCGGTGATGATCC	AATTGATCGCAGCTGTCAGC
gi646098128gbKL023721.1_352130	wft4_QDD_S05	non-amplification	AGAT	GCTTACCGCCACTACCACAA	CTCAATCTACACGGGCGGT
gi646098154gbKL023695.1_3365573	wft4_QDD_S09	polymorphism	AGGC	GGCCGATGATTGTGCAAACA	CCGCATGCTAGCAATCCACT
gi646098138gbKL023711.1_1811766	wft4_QDD_S13	polymorphism	AACC	TGTGCGGTTTCATGCAAAGG	CGAGACAAACGGGTGGATGA
gi646098082gbKL023767.1_656677	wft4_QDD_S15	low-success-rate	ACAG	ACTATTGCTGTAATCACACGCA	TATCTTTGCTGTACGGTTTCGC
gi646098075gbKL023774.1_558577	wft4_QDD_S16	polymorphism	ACAG	CAAGCCACTCCCAGGAGATG	GACAGACGACATGACCTCGG
gi646098129gbKL023720.1_1943371	wft4_QDD_S17	polymorphism	ACAG	GACCGTCAACGTGGACCC	CCGACTGGACTGCTACTGAC
gi646098080gbKL023769.1_950937	wft4_QDD_S19	non-amplification	AGGG	TGATGGTGACGACCTGCAAA	GCGGATCGCGGATATTAGCT
gi646098136gbKL023713.1_273035	wft4_QDD_S22	polymorphism	AGAT	CGTTACCGATGTGCCACGTA	ACCTAGTGGATCCCTCGAAAGA
gi646098149gbKL023700.1_102593	wft4_QDD_S25	low-success-rate	ATCC	CCCGAGATGTCACCCGATG	TGTCTTAGGCAATGCTCTCCA
gi646098106gbKL023743.1_1395123	wft4_QDD_S26	polymorphism	ACAG	TTAACGGCGGTCATGCTTCT	AATGCGGCGCTTCGTTAGAA
gi646098137gbKL023712.1_2612131	wft4_QDD_S29	polymorphism	ACGC	CATCACGACAACAATGCCGG	AGCGTCATTATACCGGTGCC
gi646098104gbKL023745.1_1063884	wft4_QDD_S30	polymorphism	ACGG	TGTAGTAGGCGGGAAATGATGA	GAGTGTCGCAGCAGAACTCT
gi646098133gbKL023716.1_2516373	wft4_QDD_S31	polymorphism	ACTC	ATCACTTCGCTAGCACGCTC	AGTTACGTCGTTCCGTGTCC
gi646098120gbKL023729.1_1886803	wft4_QDD_S32	polymorphism	ACAG	GTCTCGGTATGCGTACAGGC	ATTTCGATACCAGGCCGTGT
gi646098131gbKL023718.1_2165930	wft4_QDD_S34	polymorphism	AGAT	GCTGCACGCTAAGTTCACAC	GTTGCAGCTCTTCTCACCTG
gi646098127gbKL023722.1_1701193	wft4_QDD_S36	polymorphism	AAAG	CCGGCAGCACGTTTATCAAA	TTGCGGTTGATTCGTTGCAT
gi646098152gbKL023697.1_3326664	wft4_QDD_S37	non-amplification	AGAT	TTCTCTAGCTGTGGTGCCCT	CTCTGCGTGGTGGGAAGAC
gi646098122gbKL023727.1_499999	wft4_QDD_S41	non-amplification	AAAG	TTGCTGACTTACGAGCTGCT	AACGAGTCAAGCCATAGCCG
gi646098094gbKL023755.1_1175614	wft4_QDD_S45	polymorphism	ACAG	ACCCAAATACGGCAACCAAC	ATCGGTGCACAATCAGACGG
gi646098103gbKL023746.1_468775	wft4_QDD_S49	non-amplification	ACTC	GCTTCGCGTAGTCTTCAAAGC	CTGTAAATTCCAGCGGCCGG
gi646098135gbKL023714.1_1043044	wft4_QDD_S50	polymorphism	ATCC	CCTTGCACGCTCTGATAGGT	TCCCGTAGTTGGCCAAATGA
gi646098150gbKL023699.1_3290479	wft4_QDD_S51	non-amplification	AATG	TGGGAGAGTAACACGTGTGC	TTTGCGACTCCTGATGCAC
gi646098110gbKL023739.1_1872761	wft4_QDD_S52	polymorphism	AAAC	AGGGCGTTGATGTTGAGGAA	CGGCGTGATCTAGAGGGTCT
gi646098115gbKL023734.1_1169848	wft4_QDD_S54	non-amplification	AAAC	ACCACCCTCTAACCGGAAGT	GGCACGGTCTAATCGCTGG
gi646098072gbKL023777.1_303546	wft4_QDD_S57	polymorphism	AGAT	GACGGAGAGGGATTCGTCAC	GCTGCTCATGCGACAAATGA
gi646098155gbKL023694.1_2193480	wft4_QDD_S58	polymorphism	AGAT	AAGCCGAATGGGAGACACTT	ACACGTGAACAGCGTATAGGT
gi646098096gbKL023753.1_196943	wft4_QDD_S59	low-success-rate	AAGG	GGCTCTATGGGTGACGCAA	CTCCGCCATTAGGAACGGG
gi646098155gbKL023694.1_4582251	not used in this study	-	AGC	CGACCTTTCAGTGACTGGCA	TAGCCAGCCATAGCCAACAC
gi646098155gbKL023694.1_3859758	not used in this study	-	AAC	TCCTATGCTCTTCTGGGCCA	TGCTTTCGGATCAGTCGAGG
gi646098155gbKL023694.1_3569124	not used in this study	-	AGC	ATGATCATGTCGGCGAGCTC	GAGGTGGACGTCCGTGGA
gi646098155gbKL023694.1_2363479	not used in this study	-	ACC	AGCCAGAGAAACCATGCCAA	TGTAGGTGTATCGGCGCAAA
gi646098154gbKL023695.1_4391546	not used in this study	-	AGC	CCTGGAGGAGTTGGAGGGA	ATTCGAAAGCAGCCACTCGA
gi646098154gbKL023695.1_378603	not used in this study	-	AAG	CCCTAGTAGGAGGCTCTGCA	AACGACTCTGTATCGGCTGG
gi646098154gbKL023695.1_2593014	not used in this study	-	AGG	ACCTGTTGGCGGGAAACTTG	CGGGATGAATTACGCAAGCG
gi646098154gbKL023695.1_1358510	not used in this study	-	AGG	ATTAAAGAGGAGGGTGCGAGG	TTATTCTGAGCGGTCCTTGGTC
gi646098154gbKL023695.1_1248058	not used in this study	-	AAG	GCAAACGGCAAGACGGAGTA	CGCTCGACACATGCACTACA

gi646098153gbKL023696.1_3832024	not used in this study	-	AGC	TCCAGCTCAGAGGATTTGCC	TATCGTCTGTTTGGCACACG
gi646098153gbKL023696.1_3025515	not used in this study	-	CCG	TCCGTCACGATGAACACGAC	AGCGTTGCTATGGCTCGAAA
gi646098153gbKL023696.1_2251340	not used in this study	-	AAG	GTGGTATTGGTATGGCCCGA	TTTAGTCCTGTCCTGCGACA
gi646098152gbKL023697.1_4413044	not used in this study	-	AAG	GTGATAGATGGTTTCCAAGGCC	CACGCCGGGCCGTTAGATA
gi646098152gbKL023697.1_3455764	not used in this study	-	ATC	GGAGATTTCGTGGCCGTGTA	TGAACAGCAGAGCGTGAAAT
gi646098152gbKL023697.1_2633263	not used in this study	-	AGC	GCCGGTAGAAGAGCAGTAGC	ATCACGCCGGTCATCACATC
gi646098152gbKL023697.1_1735260	not used in this study	-	AGC	CCCTTTGTCCACTGTGAAATCC	TGCATTATGTCGTTTGAGCAGA
gi646098151gbKL023698.1_2432458	not used in this study	-	AGC	TGGCGGCGCTGTGAATTT	AGTCCCTATACTCGTCGCGA
gi646098150gbKL023699.1_3874696	not used in this study	-	AGG	TATTCACCCACAACACGCGG	CGAGTTTGTGAGTGAGGCTCT
gi646098150gbKL023699.1_3707050	not used in this study	-	AGC	CAACGCAAACACCCTGCAG	GAGTTGGCGCATCCCATACG
gi646098150gbKL023699.1_3623744	not used in this study	-	AGC	GTGCTTTCAGCCGGCATC	TGCACATTTGTCACCTCGGT
gi646098150gbKL023699.1_2143947	not used in this study	-	AAC	CCTGTGGCAGGTCCGATC	TACAGCATACTTCGGCAGGG
gi646098149gbKL023700.1_931942	not used in this study	-	AAC	CTGCTGCGTCCTGAGAGTG	ACCGTCGCACCTGCATTG
gi646098149gbKL023700.1_613065	not used in this study	-	ATC	CAGGTTCAGGTAGCTGGAGC	GTTCTGGTCGGCGAGTTACA
gi646098149gbKL023700.1_3561595	not used in this study	-	AGC	TGCCTCTTGTCTAGACCGGG	GCAGCTCCAGCCCGAAAG
gi646098149gbKL023700.1_2539789	not used in this study	-	AGC	CCGATCAGTGGCCTCTTCTC	TTTCAGTTTGAGAGCCGGCA
gi646098148gbKL023701.1_3299208	not used in this study	-	AAG	GCTCGAGCACCACACTATACC	TCCATGTTAACCAATGTGTCGT
gi646098148gbKL023701.1_1185884	not used in this study	-	AGG	CAGCCTGACAAAGCACTGAC	TCCCTGAAATGCTGCTCCTC
gi646098145gbKL023704.1_377728	not used in this study	-	AAC	AATGTTATGCACCGCTCTGC	TACGCGGCGACATAATGTGT
gi646098145gbKL023704.1_3345810	not used in this study	-	AAG	CCGTTGTGCTAGTTCACGTG	GTCGTGACCGAGAGTGACAA
gi646098145gbKL023704.1_26027	not used in this study	-	AAG	CAGCAATAAGGCAGCACCCA	TTTCTCATACCCACTGGCCG
gi646098145gbKL023704.1_2029117	not used in this study	-	AAT	GAGGCAGTGAAGCAGAGGTT	GCTAGTGGTGGTATGAGGCA
gi646098144gbKL023705.1_449684	not used in this study	-	CCG	GGAGCGCTGCAATCATTTCC	TTCTCGCGTCAAACAGGTGT
gi646098143gbKL023706.1_940540	not used in this study	-	AGG	GCCAGAGTCAGCTTTCAGGA	CCAACTGGCTTATTACCCACCT
gi646098140gbKL023709.1_222825	not used in this study	-	CCG	GGGCGTTTGAACCTTTGGTC	ATAGTAAGCATCGCGGCTGT
gi646098139gbKL023710.1_656779	not used in this study	-	AGG	CCTGCGGACACGACACAATA	GTTCCTGAGGTTCCTGGTGG
gi646098139gbKL023710.1_2623524	not used in this study	-	CCG	CGCCTCTGACAATGATGGGT	CGTTACTCAAGGCACCTCGT
gi646098139gbKL023710.1_2195000	not used in this study	-	AGC	GGAGTGACGAACAGGACGG	TCTACAAATAACAGACTGCGCA
gi646098138gbKL023711.1_915507	not used in this study	-	CCG	CCTCCAGAGCAAGGACTTGG	GCGCTTGTAGATATGCGTGC
gi646098137gbKL023712.1_2505495	not used in this study	-	AGG	ACCTCTACTATTACGGGCTCG	AGGGAGAAAGAGGGCGAAGA
gi646098137gbKL023712.1_1928750	not used in this study	-	AGC	AGGAGAAACGGGTGGTGATG	CACCTTCGTGCTTTCCAACG
gi646098136gbKL023713.1_1402286	not used in this study	-	ACT	GCCGACTCAACGAAATGCAA	CAGAGCACTATAGGCGGCAG
gi646098136gbKL023713.1_1113639	not used in this study	-	AAG	TTACAAGCAGACGAGAGCCG	ATACCAGGTGCACCGAAGAC
gi646098135gbKL023714.1_2192038	not used in this study	-	AGC	TATCAACGCTCACCTACGCC	GTCGCTCCCGCCCTAATTTA
gi646098135gbKL023714.1_152143	not used in this study	-	AGC	GGAACCCAATGCGGAATAACA	GAATGTCCAGGCAGCCGTA
gi646098134gbKL023715.1_339111	not used in this study	-	ACC	CCCGACTGAACCAATCTCCC	GTCCCTGCGATACGGAACTC
gi646098134gbKL023715.1_300169	not used in this study	-	CCG	TATTGACACAGCGAGGCTCC	CAATCCCATCACCTGGTCCG

gi646098134gbKL023715.1_2380899	not used in this study -	ACC	ATCGCGCTGTAAACAAAGGC	TGCGCAAGTATATCGGTCGT
gi646098134gbKL023715.1_1401975	not used in this study -	CCG	TGGCCGCTCATCTGGTTTC	ATACGACCGACATCTGACGC
gi646098134gbKL023715.1_1232893	not used in this study -	AGG	CCGCTGGAGCCCATAGTTTA	GCGTTGGCATGTAAGTTGCA
gi646098133gbKL023716.1_428116	not used in this study -	AGG	GAGTTGCACGAGCTCGAAGA	ATCAATGGAGTGGGAGAGCC
gi646098133gbKL023716.1_2626031	not used in this study -	AGC	ATATTGGGCATGCCTGGACC	GCCTCACAGCCAGTCACTAT
gi646098133gbKL023716.1_2425769	not used in this study -	AAG	CAAACCCGAGGCGAAGAGAT	AATGCGGCTCTTCCTCTTCC
gi646098133gbKL023716.1_1833084	not used in this study -	AGC	GTACCACCATGCTGACTGCT	CGTAAGGAGCCGGCATGTAT
gi646098133gbKL023716.1_1659030	not used in this study -	ACG	GACAGAGACACGCGCTTTCA	GCGGTTGAGTGAGTGCAATC
gi646098132gbKL023717.1_109937	not used in this study -	ACC	AAATTGGTACGGGTCCAGGTC	AAGCGATTACAGAGCCAGGG
gi646098131gbKL023718.1_130499	not used in this study -	AAC	CCACCACGCACCTATTCGTA	TACGTCGCGACTCACTAGCT
gi646098131gbKL023718.1_1118294	not used in this study -	AAG	TTCTGATCGACAGCGGACAA	GGTTTAAAGTCATCGCCGTGC
gi646098130gbKL023719.1_499747	not used in this study -	AGC	AAGTCTCGTTCCAGGGAGGT	TACCCACAGTGAGTCTTGGC
gi646098130gbKL023719.1_317344	not used in this study -	AAG	TCACACCATTACCACCTCGC	CAGCGAAGACGGATGCTGTA
gi646098130gbKL023719.1_306555	not used in this study -	AGC	TGTGTTTGTGCTGCGATTGG	AATACTGCTTGGCGAACGGA
gi646098130gbKL023719.1_1233324	not used in this study -	AGC	ACCGCGAGTTCTTTAGGACC	CGCGTCAGAAGAGTGTCAGG
gi646098129gbKL023720.1_1009865	not used in this study -	AGC	TCATCGAATTCAGCCGACGT	GACCATTGTTGAGCCGCTTC
gi646098128gbKL023721.1_469190	not used in this study -	CCG	ACTTAAACCGTACAGGCGCA	ATCCTTATCACGCGAGCCTG
gi646098127gbKL023722.1_2277923	not used in this study -	AGC	TGGGTCGAGTGATTGGAACG	CCCATCGAGGACTTTCACGT
gi646098127gbKL023722.1_1900989	not used in this study -	AGG	TTCCTCCGACCTACCCTGTT	TTTATGCATCCGAGCCGAGC
gi646098127gbKL023722.1_126168	not used in this study -	AAC	GGTGTCTTTCACTCTCGCCT	GTGTCGAGCAAACAAGCGAG
gi646098126gbKL023723.1_529337	not used in this study -	AAC	CAGGTCCAGCTCCCAACAG	GGCGAGTCACACTACTGTCC
gi646098126gbKL023723.1_358866	not used in this study -	AGC	GTCGGGCAGAAAGCTTGTTC	GCGCCGATTTGTCAAACGT
gi646098126gbKL023723.1_2202662	not used in this study -	ACC	TTGCGGTGCAATTCGATCTC	CGACCAGCCTCCCGATTG
gi646098126gbKL023723.1_1994828	not used in this study -	AGC	GGAATGTGGGAGTTGGCGA	GAGAGGTCCACACGATAATCCC
gi646098126gbKL023723.1_1773193	not used in this study -	AAC	GCAGTACACATGTCCGTCGA	GCGGCGTCCTATTTGAGGTT
gi646098125gbKL023724.1_609142	not used in this study -	AAC	GCGACGGGTAGATGTGGTAG	GTTTGAGACGGACGGGCC
gi646098125gbKL023724.1_512347	not used in this study -	AGC	GGACAACGTGAACCAAACCG	CAAGAGTAAGTCCCGCACCA
gi646098125gbKL023724.1_1910389	not used in this study -	AGC	CCCTCGGGCATTAGCTTTATGT	AGTGCGACTAAGGAGGGTCT
gi646098124gbKL023725.1_2119050	not used in this study -	AGC	TGAGGTGTTGTTACGACGCA	TTTCCTGCAGAGACTGAGCG
gi646098124gbKL023725.1_1834934	not used in this study -	AAC	TGGAGGTGTGAGAGCGACTA	AATCAATGGCTGTGTCGGGA
gi646098124gbKL023725.1_1830362	not used in this study -	AAG	TGTCGTGCTGTCAAGTTGGA	GTGTGCAACTGCTCAACACT
gi646098123gbKL023726.1_1912670	not used in this study -	ACC	CCTGTGCTGGTGGAATGACA	GAATTTCAAGAAGGCGCGGA
gi646098123gbKL023726.1_1795394	not used in this study -	AGC	CCTCGATTCGATTCGGGTGT	CCTTTACTTTGCCTCCGGGA
gi646098120gbKL023729.1_507532	not used in this study -	CCG	TACGCCGCCTGGATCGAT	GCCATGCTGTTCGACGGG
gi646098118gbKL023731.1_536737	not used in this study -	AAC	GCCAATGCAGTTGTCAGCC	TCCATGAGATGTCGCGTGTC
gi646098118gbKL023731.1_1761596	not used in this study -	AAG	GCACAACCCTCGAGTGAGTA	ACAATCGGCGGGACAACTTT
gi646098118gbKL023731.1_1573323	not used in this study -	ATC	CAAAGGTGGAACAGGTCGGT	TCAATGTGGCACAGTTTCGC

gi646098117gbKL023732.1_245508	not used in this study	-	AAG	GACTTGCGTCTGTCTGTCCA	TAGCACCAGTTGACGCTCAC
gi646098117gbKL023732.1_2161944	not used in this study	-	AGC	TCTTATAGCGCCAGGACATCA	GACGACCGTCCCGCATATT
gi646098117gbKL023732.1_1402741	not used in this study	-	AAT	TACACGGCCAAAGTGATCCC	AATGTGTTGAGGGACTGGGC
gi646098116gbKL023733.1_887183	not used in this study	-	CCG	CATGGCCGTGATGATGTCAT	TAGGTCGGATCAACGCTGC
gi646098115gbKL023734.1_831933	not used in this study	-	ACC	GCTGACCCACTTGCCTCTTT	CGCCGCAAATAGTACAACCG
gi646098114gbKL023735.1_970254	not used in this study	-	ACC	GTCCTCTACAGAGTAACCCAGA	GTACTCCACTCCAAGGGCAG
gi646098114gbKL023735.1_887737	not used in this study	-	AGC	AGTGGTCCTCTCCTCTCGTC	CAGCAGGGCGTTCACCTG
gi646098114gbKL023735.1_481914	not used in this study	-	AAC	GCCACTGTTACAAACGAGCAT	CACCTTTCACGATGGCAACG
gi646098114gbKL023735.1_340247	not used in this study	-	AGC	CGTTGACAGAAACCTGCAGC	GTTCCGCAGTCGAACAGGAT
gi646098113gbKL023736.1_810051	not used in this study	-	AGC	TTTGTCGACCAGGTTCGTGT	GAGGGAGCGTCACAGAGC
gi646098113gbKL023736.1_379442	not used in this study	-	AAC	AGTCGACACGGACGGTAGT	AGTGGAAACAACCCAACCCA
gi646098113gbKL023736.1_2058047	not used in this study	-	AGC	CCGACCACCTCAAAGGACTT	TTGGCAAGCCTTGTGAGGG
gi646098112gbKL023737.1_58623	not used in this study	-	AAG	CCCACGTGTTAAAGGCATGT	AGGTACGACAGTGTAGTTGGT
gi646098109gbKL023740.1_223983	not used in this study	-	ACC	GAAGGTCTTACTGGGCAGCC	CAGGCAGGCAGTAGAATCCA
gi646098108gbKL023741.1_425147	not used in this study	-	AGC	CTGGCCGGCAGGTAGGTG	ATACGGAGGGCCCAATTTCC
gi646098108gbKL023741.1_209181	not used in this study	-	CCG	GCCACCCGACACATTGTCTT	GGGTAAGCGTCAGTCACCAT
gi646098108gbKL023741.1_1847659	not used in this study	-	AGG	ACATTCGAGATGCACGCAGA	GCGCCTAATCGACAGAAACG
gi646098108gbKL023741.1_171517	not used in this study	-	ACC	AGGAGTGGTGCGACAAAGAG	CTTACATGTTGCGGGCGAAG
gi646098107gbKL023742.1_1134972	not used in this study	-	AGC	AACAAGGCCTTCAACCCTCC	CACCCTCACAGACTGCTAGC
gi646098106gbKL023743.1_271461	not used in this study	-	AAT	TTGGGACGCATTTGGCCTAT	TGTCGAGCCGTTACACCAAA
gi646098106gbKL023743.1_1556814	not used in this study	-	CCG	CCATCAGCGTCTAGGAAGGG	GCTGTTTGACTCCGTCTCGA
gi646098104gbKL023745.1_513340	not used in this study	-	CCG	CGCTGTGGTGTGGTAGTGTA	GCATTTGTCCGCGTCCATC
gi646098103gbKL023746.1_388868	not used in this study	-	ACC	CTGCGGACCAAAGGCTGATT	TCCAGGAGTAGACAGGGCTC
gi646098103gbKL023746.1_136396	not used in this study	-	AAG	ATAATCAGAGCGCCGTCAGC	GGACTTGACCGGCTACTCAG
gi646098097gbKL023752.1_1462372	not used in this study	-	AGC	CGCTTATAAATTGCGCCGGG	TAAAGCAATCTGGAGGGCGG
gi646098097gbKL023752.1_1385426	not used in this study	-	AGC	TATGAGCGGCAAGTCACCTG	AGTACGCACCGGCTGTTAAT
gi646098096gbKL023753.1_821305	not used in this study	-	AAC	AGCACCCAGTAAGGATGGTT	CGATCTAATTCAGCCGCAGC
gi646098094gbKL023755.1_430285	not used in this study	-	AGG	CAGCCGAGGCCATCATAGG	GCTCATGCCTGGAGGTCAC
gi646098092gbKL023757.1_303558	not used in this study	-	ACC	TTCCAGGCAAGGTGAGCAAA	AAGCCGCCACACTGAAACTA
gi646098090gbKL023759.1_68077	not used in this study	-	AGG	TGAGTAATACTGAATCGTGCGG	TGCCCACAGAGCTGCTACTA
gi646098090gbKL023759.1_1524018	not used in this study	-	AGC	AACTGGGCCGCCTTTATTGA	ACGGTACCCTGATCATGCTG
gi646098088gbKL023761.1_993159	not used in this study	-	AAC	TGCCAGAGTGTCACTTCCAC	TCAGTGAATCCCACCTGCTG
gi646098083gbKL023766.1_861889	not used in this study	-	AAG	AAGGTGGAGGACGGCTAGAG	ATGAGAAGTGCCTACTCGCG
gi646098083gbKL023766.1_811849	not used in this study	-	AGC	GCTACCGTCCGAGTGACAC	GCTTGTCAGGGCCGGAAA
gi646098082gbKL023767.1_1009128	not used in this study	-	AGC	AGTAAGCCGACTGCAAGCC	CGGTCAGGTGTTTGCACAG
gi646098080gbKL023769.1_12479	not used in this study	-	CCG	TCATTTCGAAGTGCATCGCC	CCGGTGCATGACAACTCAAC
gi646098077gbKL023772.1_911882	not used in this study	-	AGC	CGATAATGTGTCGGCCCACT	CTACGCGTCCTCACCTCAAG

gi646098077gbKL023772.1_641460	not used in this study	-	AGC	GAGCAAATCGCATTTCCGGT	AAATCCAGTCGCGGGAGGAA
gi646098077gbKL023772.1_63202	not used in this study	-	AGC	TGGATAATGTCTCGCTGCCG	TATCGGACGAGACGCCCTAT
gi646098077gbKL023772.1_505009	not used in this study	-	CCG	GGGAGTATACGTACGGAGCG	AAACGGACACGACAGCCAG
gi646098077gbKL023772.1_425938	not used in this study	-	ACC	CGAGCCCGAGTTCATTGATAA	CAGGTGGACAGAGTTGGCTC
gi646098076gbKL023773.1_495267	not used in this study	-	AGC	CCCACACGGTATGTTTACGGA	AGTGAGACTATATGGAGCCGGA
gi646098076gbKL023773.1_176516	not used in this study	-	ACC	AGCGTACCGGAAACACTTGA	ACAGCAGCTCCATCCTTGTC
gi646098075gbKL023774.1_623015	not used in this study	-	AAG	GGACCCTGGACAGAAGACAC	CAGACTGGTTCCCTGTGCTC
gi646098075gbKL023774.1_586808	not used in this study	-	AGG	GGCCTCAGGTGTGGTCAATA	CTGCTCCCGCCTCCGTAG
gi646098074gbKL023775.1_447751	not used in this study	-	AAG	CTGGGCTCTGACGGACGT	TCATCCAAGCGCAAGGAACA
gi646098074gbKL023775.1_365215	not used in this study	-	AAG	CACTGAACCTGTCTGGGCC	ACCATGAGAACGCCTTCGAG
gi646098074gbKL023775.1_31969	not used in this study	-	CCG	TTTATACCGTCGTCCCTCGC	ATATTTCCCACGGCCGGAAC
gi646098074gbKL023775.1_176770	not used in this study	-	AAG	GCGGAGTGTACAGTGCATTG	TCGCGTTCGCTATGACTGTC
gi646098072gbKL023777.1_586883	not used in this study	-	ACC	GCTTCGCTGGAATTTCGCAA	TCGGTGCTGGGTGTTATCAG
gi646098070gbKL023779.1_663144	not used in this study	-	AGC	GTGCGCTGATACGGTTTGAG	GAGCTGGAGGAAGACAGTGG
gi646098070gbKL023779.1_242278	not used in this study	-	AGC	CTCAGGAGAGCGCTGAGATG	AGGCCTCCTTTCGAATCACG
gi646098068gbKL023781.1_75199	not used in this study	-	AGG	CGTGCGCCTGAGACGTAAT	CACGGCGACCCACAAGAAT
gi646098068gbKL023781.1_691193	not used in this study	-	AGC	GACTACGCCACGCCACCA	GCGCATGGCTTTCGAAAGTT
gi646098068gbKL023781.1_345202	not used in this study	-	AAC	TCAGTGCTGTGAGGTGACAC	GGCAAGTAGATCGTTGAACGC
gi646098066gbKL023783.1_52420	not used in this study	-	AAG	TGACTCCCACCAAAGAGCAC	GGGCCTGGGACAACCTAATG
gi646098061gbKL023788.1_717698	not used in this study	-	AGC	GCAGGAGGGCAGGAATCAAT	GCCTTGCGTTTGCCTTCTATT
gi646098061gbKL023788.1_70067	not used in this study	-	AGC	TGGATTTGACCTGCAAGTACGA	TTGCTTGAAGTTGCATTTGGG
gi646098061gbKL023788.1_203341	not used in this study	-	AGC	ATCAGGCGGAGAACAAAGGG	GCTATTTATTCGGGCAATCGCT
gi646098060gbKL023789.1_51679	not used in this study	-	AAC	AGGCGTGGACAAACTGGAAA	GGACACGCAACCACACTTAC
gi646098057gbKL023792.1_405282	not used in this study	-	AAC	CAGTTGTTCATGACGCGGAC	TAACGCAACTGCCACTGCTA
gi646098057gbKL023792.1_34603	not used in this study	-	AGC	GGGCTGTCTGTCTGATGTGATA	TTTCGGATTATTTCACGGGACA
gi646098054gbKL023795.1_472254	not used in this study	-	AAG	GGGAAGCACATTTGTTGTACCG	CTGCATGGACATGGACACCA
gi646098051gbKL023798.1_137072	not used in this study	-	AGC	AGGCGGTCTGACAGGTGT	ACGGAGCTCCACAACACG
gi646098046gbKL023803.1_402802	not used in this study	-	AGG	GCACAGTGCGAACGGACC	CCAGAGGCCGACAGCATAA
gi646098045gbKL023804.1_154041	not used in this study	-	AGC	ACGCCAGGAACTCCTTATGG	TTCGATCCCACCATAACGGG
gi646098044gbKL023805.1_730615	not used in this study	-	AAG	CGGACTCAGTTCCGACAGAG	GTCTCGCAAAGGAAACGTGG
gi646098044gbKL023805.1_526518	not used in this study	-	AGC	CTTTCACTTGGCTAAGCGGC	CTCCGAGGTGAAACCGACC
gi646098043gbKL023806.1_779044	not used in this study	-	AAG	AGCAGTGAAGGTGTTCTGCT	GCCGCCTTAACGACCAAAC
gi646098043gbKL023806.1_50768	not used in this study	-	AGC	TTGACATCTCTGCGCTGCTA	AAGTGATTAACGACGGCGAC
gi646098043gbKL023806.1_445807	not used in this study	-	AGC	GGAGGAGGTCGACGATTTCTG	TCCGGATCATCGAAGGCG
gi646098042gbKL023807.1_336213	not used in this study	-	AGC	GGCTGCTCCATAATGCATGC	CACCAAGAAGGGCTGCTACA
gi646098040gbKL023809.1_876780	not used in this study	-	AGC	TTGCCTACATGCAGGGTGAA	GCAAGCAGCCTTTGATGTCC
gi646098040gbKL023809.1_381428	not used in this study	-	AGC	TTTGAGAGGATGCGCCACC	ATTGCGTCCTCACATTGGTG

gi646098039gbKL023810.1_87918	not used in this study	-	CCG	TGTTCTTCATGCCGTCGTCC	GGCGCTGGACACCATGAG
gi646098038gbKL023811.1_286422	not used in this study	-	AGC	CTGCTCGGTCTGGCTCAAA	GGAGTTCTACTGTCGACGGC
gi646098037gbKL023812.1_545918	not used in this study	-	ACC	TAAGGGAGTCGTTGCACCAG	CAGCAACAAAGAGCCTTGGC
gi646098036gbKL023813.1_245897	not used in this study	-	AGC	GCCTATTTCGGACGGACGTT	TAAACTCGCGCCAACTCTCC
gi646098036gbKL023813.1_241715	not used in this study	-	AGC	CATATCGAGCCCTGCCCTG	AAAGGAATGTGCTCGTTGCG
gi646098033gbKL023816.1_392712	not used in this study	-	AGC	TCACTTTCTTTCGTCGGCCG	AGTCCCGCGTTTAACTTCGA
gi646098032gbKL023817.1_763618	not used in this study	-	CCG	GCACGAGCGAACGTAAACAC	TCCCATAATGAGGGCTTGGC
gi646098031gbKL023818.1_743693	not used in this study	-	AGC	GAGCAGTTTGATAGCGCCAC	TCGCCCAACCTCGTATCCTA
gi646098030gbKL023819.1_380282	not used in this study	-	AGC	TGCCTTCCCTTTGCCATTGA	GTCGACGGGTTTACGGGAAT
gi646098028gbKL023821.1_218273	not used in this study	-	AGC	ATGAGGACCGTCTTCAGCAC	GTAACTCACTACTGGCGCCG
gi646098025gbKL023824.1_68190	not used in this study	-	AAG	GCCCAATGTGACATCGAAGC	CCAGAAGTGCTCATGGCCTA
gi646098025gbKL023824.1_246187	not used in this study	-	AAC	GGTGATAGTGCTTGGGCCAA	CAGCAACCGTTCACCAGTTG
gi646098020gbKL023829.1_649087	not used in this study	-	AGC	TCCCAGGGCCATCTCAGTTA	TTAACAACCCTCGGGCGTTT
gi646098017gbKL023832.1_203788	not used in this study	-	AGC	ATGCTTGCTCTGCAGGTCC	CCGTAATCTCGTTCCGGTGG
gi646098015gbKL023834.1_283042	not used in this study	-	AAG	CGCACAAATTTATGGCGGGT	CGAAACTTCGCAGGGCTAC
gi646098013gbKL023836.1_410437	not used in this study	-	CCG	ATATGGAACGCACGTGACGA	AACTACTAGCGGGATGGCCT
gi646098013gbKL023836.1_226590	not used in this study	-	AGC	AATCCAACAGTCGGTCCAGC	CTGTGAGTCCCGTGCCTTAA
gi646098012gbKL023837.1_203711	not used in this study	-	AAC	GTATGTCGGTCGTCCTAGCG	AAATGAAACGTGGGCCTCTAGT
gi646098010gbKL023839.1_583387	not used in this study	-	AGC	CGGCGGCGTATTTCAACATT	CGAAAGCGTCTGGGAGGAG
gi646098010gbKL023839.1_25665	not used in this study	-	CCG	GGTGGTCCCTCTGTGTAATTAC	TTGCAGAGGGATTTGTCGCC
gi646098004gbKL023845.1_344619	not used in this study	-	AGC	ACACTCGTACACCAGTTCGT	CTCATGCCGGACTTGAGCTC
gi646098000gbKL023849.1_345558	not used in this study	-	AAC	AGGCAATTCCATGTTTGCTTGT	AGCCGCACGAAACTGTAATTG
gi646097999gbKL023850.1_636916	not used in this study	-	AAG	TCATAATCACCTCGCAGGGC	TTTGACCCGTTCAGCAGCAA
gi646097995gbKL023854.1_359011	not used in this study	-	AAT	GCCTAAGAAGATATGGTGTGCC	CACCTCGAGACCCACGGTA
gi646097994gbKL023855.1_23089	not used in this study	-	ACC	CCGTTCTTGCTTTGGCCAAT	CTGCTCTGGGTGTCCGTTAC
gi646097994gbKL023855.1_223340	not used in this study	-	AGC	GCATTTAGTGGCCCAGATGC	CGCCACTAATAACGCTCCGA
gi646097993gbKL023856.1_252606	not used in this study	-	AGC	CGCACAGAACGCATTTGCT	GCCTGCAGGAACCCATAGG
gi646097989gbKL023860.1_361001	not used in this study	-	AGC	AGATGGGTTTGACCGCAACC	CGCCTGATGGGTAGGCAATA
gi646097987gbKL023862.1_647323	not used in this study	-	ACC	CTGTCAGTTCACCAGGGAGT	CTTGGTTGCTGGTTTGGCTT
gi646097985gbKL023864.1_431911	not used in this study	-	AGC	CTCGCTCTCCGCTGCCTTT	GCAGGAAGCCGTTTGCAAG
gi646097984gbKL023865.1_57124	not used in this study	-	AGC	CCGCTTCATTTGGACGCTAT	TAGTTAGATGGCTGCCGCAC
gi646097982gbKL023867.1_326191	not used in this study	-	ACG	GGAGTGAGCCATTCCGCTAC	TGCTCTGAAATGAAGACAGCC
gi646097980gbKL023869.1_457552	not used in this study	-	AGG	AACATTCAGCACCGGACTCA	ATTAGTCACTGCTCCGTCGC
gi646097979gbKL023870.1_35789	not used in this study	-	CCG	CAATCGATGCTTGCAGACCG	CCGTATGTGTTTCGCAGGTG
gi646097978gbKL023871.1_160821	not used in this study	-	AGC	GATGGCCCAATGTGTTGTCA	TCGGAAAGTGAATAGCGATGC
gi646097976gbKL023873.1_242533	not used in this study	-	AGG	CGATTGATGAGCGTCGTGTC	ACACGAAAGGAACACAGGCT
gi646097975gbKL023874.1_424551	not used in this study	-	AGG	GGATGGCACGAACCCTGTAA	CAGACTCTTACCGCGCTACC

gi646097974gbKL023875.1_16833	not used in this study	-	AAG	AATCACGACGATGCGAGAGT	GACAGTAGAAGCCTGGGCAA
gi646097970gbKL023879.1_355582	not used in this study	-	AAG	TGAGGTTTCAAGTGTTGTGGAA	AAGGCGTCGGGAGGTTTAAC
gi646097970gbKL023879.1_131441	not used in this study	-	AAG	TCACTTACCTCAGGAGACACG	CTGCCTCATTGAACTATGTGCA
gi646097960gbKL023889.1_141001	not used in this study	-	AAG	GGCGCTAAAGACCTAAGTTGC	CGTGATAACGGGTCAAACAACA
gi646097955gbKL023894.1_440948	not used in this study	-	AGC	ATACGGTGAAACAGAGCGCA	ACTGACGTTCGTGGGCCG
gi646097955gbKL023894.1_191050	not used in this study	-	AGC	GAGCAGCACTCCAGCATTG	TATGGCCACTAGCTCACGTG
gi646097947gbKL023902.1_110549	not used in this study	-	CCG	GGATCGCCGAAGAGCAGAG	TACCAAATCGCCTTCCGTGT
gi646097926gbKL023923.1_74027	not used in this study	-	AGC	GAAAGGACTCCGACCGACAG	CACTCCACTCTCGTCGTCAC
gi646097922gbKL023927.1_292161	not used in this study	-	ACG	GCGCAGTGTAGATTAAGGCC	TCCGCTGCTTGATACAGTCC
gi646097922gbKL023927.1_239104	not used in this study	-	AAG	GTCAGTGGGTCGACCTTAGC	CTGTGGGCCGAGTAGAAAGG
gi646097919gbKL023930.1_336968	not used in this study	-	ACC	ACTGTCGCACGTTTAGCGG	AGTAATTCTCGAGGCCGCAG
gi646097903gbKL023946.1_73944	not used in this study	-	AAG	GACGAGTAACCTGGTTATCTGC	CATGGTCAATGCAGCACACT
gi646097898gbKL023951.1_352141	not used in this study	-	AGC	GGCCAGCCTTTCACCAGAAA	CACCGCACACCACGTTCAG
gi646097891gbKL023958.1_327674	not used in this study	-	AGC	TCCCGCAGACGAATCGAATA	GCTCTAGGCTGCATGCGAT
gi646097891gbKL023958.1_103281	not used in this study	-	AGC	GCAGCAAGTTCAGTGCAGTG	TAAACCTGCTCCGCCAACAT
gi646097875gbKL023974.1_79605	not used in this study	-	AAC	GCCAGGACGTTGATTTGCAC	ACAGCTGTCGATCACCAAAGA
gi646097867gbKL023982.1_154891	not used in this study	-	AGC	TGAGCCGCCATACAGCAG	GGAAACACGCCGACAGAAAT
gi646097862gbKL023987.1_145487	not used in this study	-	ACC	CACGACGAGCTCAACTGACA	CGACGAGGGCCGATGTTTA
gi646097858gbKL023991.1_82939	not used in this study	-	AGG	TGTCCTGTGGCTCGTTATCT	GGCCAAGCATTTCCGAAACC
gi646097845gbKL024004.1_275875	not used in this study	-	AAG	CGCTCTGACCTGCTCTGTTA	CCTCATTAAGGTGGCCTGCA
gi646097842gbKL024007.1_163567	not used in this study	-	AGC	TGTGGCGTCGTGGACTTTAT	AACTAGTACGTGCTGCTGGC
gi646097835gbKL024014.1_265060	not used in this study	-	AAG	CCGCATTCAATTACAGTGCGT	CTTTGCTGCTGGGCTTGTG
gi646097829gbKL024020.1_202153	not used in this study	-	AGC	GGGTCTACATACAGCGAGCG	AACAGCTTGACGACAGACCC
gi646097827gbKL024022.1_37136	not used in this study	-	ACG	CCGCGTCCCAAAGATGAGTA	AGGGACCTGCCAGAAATTCC
gi646097821gbKL024028.1_191594	not used in this study	-	AGC	CAGTCCTCAGCTCTTGTCGG	CTTGCTGATGTTGGCGTTCG
gi646097821gbKL024028.1_119125	not used in this study	-	AAG	CGTCCGCTACTCACAGTCAG	CGTGTCGCTGGTGGAGATAA
gi646097817gbKL024032.1_68140	not used in this study	-	AGG	ATGTCATGCCGGCAGATGTA	CCGCGCACTCTCAATTTGTC
gi646097815gbKL024034.1_120209	not used in this study	-	AGC	CGGCGTGCGAATCATTTACC	CCAGCAGGGTAGACTTTGCA
gi646097813gbKL024036.1_111608	not used in this study	-	AAG	AGCGGTGAATCGTGGACAAT	TTTCGGAAACACGTGGGAGG
gi646097783gbKL024066.1_99078	not used in this study	-	AAG	CGGTAGGAAGGAAGATGGCG	CAAATGGCGGTGAGTGGTTG
gi646097783gbKL024066.1_146693	not used in this study	-	CCG	TCCATCCCTCGGTCAGCTAA	AGCGTCACATTATGTCCCGG
gi646097774gbKL024075.1_96546	not used in this study	-	AGC	TGGGTATGGCAAACCCTTGT	CCATACCGCCCTCTTCTGTC
gi646097774gbKL024075.1_22227	not used in this study	-	AAG	GAGCCAAGGCAGTGTCAAGT	CTGTGCTCAGCTCACCGATT
gi646097742gbKL024107.1_219249	not used in this study	-	CCG	GTCTATTCTGCCCGGGCTC	AAGATCGCTGTGCACGAATG
gi646097742gbKL024107.1_118708	not used in this study	-	AGG	CGCATCTCGAGCGGACTT	GTCCGCTACGACGAGCAC
gi646097733gbKL024116.1_160209	not used in this study	-	AAG	CGCGTAGGCCGATGATAGAA	GCTGTAGCCGTTTCACGTCT
gi646097728gbKL024121.1_220144	not used in this study	-	AAT	GTCCTGTTGGTACACAAAGCC	GGATGCAAGTAACTTCCCAGC

gi646097716gbKL024133.1_77964	not used in this study	-	AAT	ACTCGGCGACAACGGTAC	ACGTCCTGTCGTATTGTGTAGT
gi646097683gbKL024166.1_97964	not used in this study	-	AGC	GACACCCGTGACCTCTTGAC	CCTCTTGTTCTCCACACGCA
gi646097679gbKL024170.1_1665	not used in this study	-	AGG	TGTGAACCTCCTCAACAGGC	GAGAGCCTGGAGAGCGATC
gi646097663gbKL024186.1_87886	not used in this study	-	AAC	ACAGAGTGACAGCCGTCTTG	ATTTGTAACGTGCAAGGGCG
gi646097652gbKL024197.1_19583	not used in this study	-	ACG	ACACGCGACACTGGAATACA	GTCCACTGTCCTGTCGAGTC
gi646097629gbKL024220.1_70694	not used in this study	-	CCG	CTCTTCCTCCGACGCGTAAA	ATCTTGTCTCGCCCGGATTG
gi646097620gbKL024229.1_177	not used in this study	-	AGC	CACTCTCCTGCTCCTTTGCA	TCTCCAGAGAAAGCCACGTG
gi646097609gbKL024240.1_60079	not used in this study	-	AGG	CTGTCAGGGAGGACGCTTATC	AAACAACTTGGAAGCCTGCG
gi646097558gbKL024291.1_26068	not used in this study	-	AAG	CCAGTTCGATGTGGTCAAGG	GCCTTTCAGACAACATCGCC
gi646097532gbKL024317.1_16561	not used in this study	-	AGC	GACTCACCCTCCCAGTGC	GGATCGGGTCGTTGAGGAAT
gi646097488gbKL024361.1_16152	not used in this study	-	AGC	CAGTGGCTGCATGGGAGC	GTGGGTTTGAAAGCTCTGCC
gi646097472gbKL024377.1_1720	not used in this study	-	AAG	AAGACGACGCTTACAGACGG	GTACTGTCGTGCGGACCTTT
gi646097396gbKL024453.1_27517	not used in this study	-	ACC	GTTGCTGACGTCACAAGGC	CTGTGATTGCTAGCATGTCGC
gi646097367gbKL024482.1_19147	not used in this study	-	AAC	ACATGGGTTCGCTTAGCAGT	ACGTCCCTTTCCGGAGTAGA
gi646097298gbKL024551.1_22388	not used in this study	-	CCG	TTGTTCTGCGTCGCCAGAAT	GGTGAACAACGGCGGGTA
gi646097056gbKL024793.1_6383	not used in this study	-	AGC	GGGCAATTAGTCCGTTCTCTTG	GGGCGTGTCCTGTCCACC
gi646096999gbKL024850.1_5805	not used in this study	-	AGG	CCAGTGACTGTGTCCTTCCG	CCGTATGCATGGCTCATGTC
gi646096978gbKL024871.1_24111	not used in this study	-	AGC	CAATGTTGCCGAGTTGCGAA	CTGAAACGGCCCGAATGTTG
gi646096967gbKL024882.1_2833	not used in this study	-	AGC	TATCGTATGGCGGGACAACG	GACTCATGCCTGCTCTGGAA
gi646096956gbKL024893.1_4736	not used in this study	-	AAG	GGTGCAACGATCACAGTTGG	AAGAGCCCGAAATACGCTCC
gi646096859gbKL024990.1_4383	not used in this study	-	CCG	TGCGTGTTTCTGAGACTGCC	CTACCTCGCGTCGCCTGG
gi646096750gbKL025099.1_7630	not used in this study	-	AGC	TAGTCTCGGCGGCACTTACT	TTGGAATTCTTGCGGCGGC
gi646096670gbKL025179.1_20308	not used in this study	-	AGG	AAGAGATTATCACGCGGCCC	ACGCGATGTAGCAGGCTATC
gi646096488gbKL025361.1_10652	not used in this study	-	AGC	AGCGAGAATGTCAATCGGCT	GCACGTCGACAAGATGGAGA
gi646096372gbKL025477.1_1	not used in this study	-	CCG	GCAGTGCTAATGACGAGGCA	CGCCGTTTGAAGGAGCTTC
gi646096189gbKL025660.1_2266	not used in this study	-	AGG	AAGGCGTCGTTCATAAAGGG	CTTGACCCTGGAGTTCCCAA
gi646096170gbKL025679.1_11283	not used in this study	-	ACG	GCTCAGTTCCAGTCAGTCGT	ATACCGCTTGACGTCACGAC
gi646095962gbKL025887.1_1478	not used in this study	-	AAG	TGCAGTGTGACGACTGTTCA	AATGGCCCGCATTAATTGGC
gi646095960gbKL025889.1_8924	not used in this study	-	AGC	GGCTGCAGGGCTATCTGTTA	AGGAGATGTGACCAACGCTG
gi646095885gbKL025964.1_8997	not used in this study	-	AAG	ATTTGAGCCCACATCCCGC	TAGTGCGTGATTTAGGGCGG
gi646095711gbKL026138.1_471	not used in this study	-	AGC	GTCCCTGAGTACCAGCCATG	CGCCACAATTCGCAACAACT
gi646095659gbKL026190.1_6980	not used in this study	-	AGC	GATACGCAGACCTGGTCGTC	GCAATTTGCTTCGGTCGGG
gi646095609gbKL026240.1_11405	not used in this study	-	ACG	AACTAAGATCAGGTGCGCGA	CCGAGACGGTCATATCTCTGA
gi646095570gbKL026279.1_2549	not used in this study	-	AGC	CTGTCCCAAAGGCTTAGGGC	CATGCCACACCTCGGTCATA
gi646095239gbKL026610.1_4499	not used in this study	-	ACG	GTGACGCTTTCTTCCGTGTG	AGCATCCTTGGTGTGGTCTG
gi646095124gbKL026725.1_5291	not used in this study	-	AGC	GGTCGATGCAACAGCTCTCC	TCCGGGTTCGCCAATAACAA
gi646095089gbKL026760.1_6859	not used in this study	-	AGC	CAATACGCTGGAGTCCCGTT	GAGCAGACTTCTCCAGCCTG

gi646094729gbKL027120.1_2743	not used in this study	-	AAG	AGGAAGGAGGCTAGAGACGG	CGGCAATGTGAGGTGTGACA
gi646094389gbKL027460.1_8605	not used in this study	-	AGC	GGGATGAGTCGCAGGATTCA	GCTGATTGACAATGGCTGCG
gi646094373gbKL027476.1_9355	not used in this study	-	AGC	TGTCATCCACATCCACGCTC	CGTCAACGGGCGCCAAAG
gi646094032gbKL027817.1_219	not used in this study	-	AGC	GCGTCACACGTCACAACAAG	AGCCAGCCGCCCAATATTTA
gi646098155gbKL023694.1_807283	not used in this study	-	AAAG	CAACTCCCTCGCCGTCAG	TGAACTGTGTCGGACGTGG
gi646098155gbKL023694.1_2884491	not used in this study	-	ACAG	GGCCGGAAGCATTCTTGTTG	GCACTCTGAGGGAACGAGTG
gi646098155gbKL023694.1_2279227	not used in this study	-	ACAG	GTGGCCGACTAGCTCATCTC	ATTGCCATGGACGTGGACAT
gi646098152gbKL023697.1_3389552	not used in this study	-	AGGC	GGCGATCGTGGCGGAAAC	TTGACGCCTCTGATAAGATCGC
gi646098152gbKL023697.1_1671157	not used in this study	-	AGAT	TCGACCATGACAACCGACTG	TCCGCTTTCTCTTGAAGGACA
gi646098150gbKL023699.1_3887864	not used in this study	-	ACTG	GTTTGCAACTCCGCGACTC	AGAGGGTAAACTTGGGATGGT
gi646098149gbKL023700.1_940743	not used in this study	-	ACAG	CGCCGCTACAACAAGAGACT	TGGTCGGGTGTGAAATGTCA
gi646098139gbKL023710.1_357084	not used in this study	-	AGCC	TTTCGTGTCCAAGGTCCAGG	TGCAAGCAAGTCTTCTACGTCT
gi646098138gbKL023711.1_987747	not used in this study	-	AAAG	TAGGAGCAAAGCACCGTTGT	GTCACCCTGCCACTCACATT
gi646098135gbKL023714.1_3264964	not used in this study	-	AGAT	CAGCAACTTAATGGGTGCGG	ATGTGAAACACGAGCCAGGG
gi646098133gbKL023716.1_1223042	not used in this study	-	ACGC	TACCCTTGTCGGAGCAACTC	GTACCCAGCGAGGCACTAG
gi646098131gbKL023718.1_1617031	not used in this study	-	ATCC	TGGCTAGATGGCTAGATGGC	TCCATCTGGCCTTCTTTCCA
gi646098122gbKL023727.1_1151296	not used in this study	-	ACAG	ACGGTGACTGACTTGTGCTT	TTCGTCAGCACAGGGTGTAC
gi646098120gbKL023729.1_1676102	not used in this study	-	ATCC	CACCCTCGGCTGATACAGAC	GAAGTTTCGAGCCCTGTGGA
gi646098115gbKL023734.1_833024	not used in this study	-	AATG	ATGAGTGGAACGAAGCTCGA	TGCCATTGCTCAGTGTAAGACT
gi646098110gbKL023739.1_120938	not used in this study	-	ACAG	GCATGGTAGCGGTGTTCTTG	CAGACTCCGCAGTGCCATTA
gi646098106gbKL023743.1_703583	not used in this study	-	ACAG	ATTGCGCTGGTGGTTACTCC	ACGCCAAATTCACTTGCCTA
gi646098106gbKL023743.1_263249	not used in this study	-	ACGC	GGTGGGAAACAGTAAACGCG	GGTGTACGTCAGTAGGCTCG
gi646098106gbKL023743.1_1206152	not used in this study	-	AATG	TCGCCCGGATGATTCTAACC	TCTAGTGCGTCGATCGGTTC
gi646098068gbKL023781.1_94532	not used in this study	-	AGGG	TCGGAGCAAGCGTCAGTTAG	TGCATCGAACGAACAGGTGA
gi646098067gbKL023782.1_317522	not used in this study	-	ACGG	CGTACAGTACACCACCGACA	TGATTGAGAGTCGAGCACACT
gi646098067gbKL023782.1_279465	not used in this study	-	AGGG	GCAGGCAGCTCGTTACGATT	TGGCATCGTTATCCTGACAGG
gi646098059gbKL023790.1_714776	not used in this study	-	ACAG	TTTGCACTTCATTTGGCCGC	CTAGGCGACCATGGCGTTAA
gi646098056gbKL023793.1_46765	not used in this study	-	ACGC	ACCGACCACCGTTGAAAGAG	TCCACCAGTCGTTTGCCTTTA
gi646098022gbKL023827.1_37076	not used in this study	-	ACTC	CAATCTCCGATTCCAGGCGA	CCGGCCGAGTGAAAGTAGTT
gi646098020gbKL023829.1_234701	not used in this study	-	AGAT	TCGCTGACGCTGTTCTGATT	CGAGAGCACACGGTCACG
gi646098020gbKL023829.1_218711	not used in this study	-	ACTC	TAGACCGCCAACGTTTGTCA	GCTGGAGTAACGTGAGCAGT
gi646098019gbKL023830.1_586533	not used in this study	-	AGGG	CACATTAGGCTCGACACGCT	CTGTAGGCTGTACGGCGAG
gi646098016gbKL023833.1_590560	not used in this study	-	AGAT	AGCCAGGAGTTCCAGGAGTC	TCCGCCACATCACCCTAGTA
gi646098015gbKL023834.1_76336	not used in this study	-	AGAT	TATTGATGAGTAGCGCGGCG	TCACTCCCATTCATCTCGGC
gi646098014gbKL023835.1_493141	not used in this study	-	AGAT	GCGGCAATCGGATTTCCATC	TGGGATGTGGGCACAATCTT
gi646098007gbKL023842.1_749726	not used in this study	-	ACAG	AGCCTGTTCCTCTTTGAAGCA	CGTCGGGAGCATTATGGTCA
gi646098007gbKL023842.1_274413	not used in this study	-	ATCC	GGGCTTCAGTTCCAAAGAGGT	TTTCGCACCTTGTCCTCGTT

gi646097996gbKL023853.1_484025	not used in this study	-	ACTC	GAACTGGCGAAAGTACACGG	GTCCTTGCAGTCGTCCGT
gi646097980gbKL023869.1_365740	not used in this study	-	ATCC	CCTGTGCAGTAACCCTCGTT	GCCGTTTAGGTCTTTGGAGGA
gi646097960gbKL023889.1_316498	not used in this study	-	AAAG	CCCGCTGAAGTCGGATCTAG	TCATCAACAGCGGAACCCTC
gi646097930gbKL023919.1_278984	not used in this study	-	ACAG	ACATGCTTGTCCTGCGACTC	TACAGCTAGCTCGTCACAGC
gi646097891gbKL023958.1_296111	not used in this study	-	ACAG	TAAGCGCTGCACTATTGGCT	CTACCGATCGACCTGCCTTG
gi646097850gbKL023999.1_281364	not used in this study	-	AGAT	TATTATTTGCTGCAGGGCGC	CAGTCACTCGCATTAACGCC
gi646097808gbKL024041.1_243041	not used in this study	-	AGGG	CGTGAGTGCTTGGCGATTAT	AGCTCTGGTTGCTTGCAGTA
gi646097791gbKL024058.1_118879	not used in this study	-	AGGG	CTCTTTCCAGGACGCGTGTT	GGCCAGCGGAAAGAGACC
gi646097628gbKL024221.1_20629	not used in this study	-	AGGG	AAGTCAGACAACAGCGTGGT	TTGCTGACCGCTGGATAGC
gi646097391gbKL024458.1_18713	not used in this study	-	ACGC	TGAACCCGAGTAGGCACAAG	GGAGTATTTACGTCAGCCGC
gi646097190gbKL024659.1_20720	not used in this study	-	ACAT	TTCACGCGGTGTTTCGTAGT	CTTTCGGGCAGAGCGAGTAC
gi646097126gbKL024723.1_7676	not used in this study	-	AGAT	CCACCCTGACAAACGCAAAG	CGATAACGGACCACTCGGG
gi646097056gbKL024793.1_15663	not used in this study	-	ACAG	TTGGTGCGGACATACTCGAC	GGCCTTGGCGATCTACCTAC
gi646096742gbKL025107.1_22446	not used in this study	-	ATCC	GTTGTTTCGGAGCTGTTCGC	TAGCCGGCGGTGCTGATT
gi646094618gbKL027231.1_1422	not used in this study	-	ACAG	TCGACCTTGACTGAGTCCAC	GCTCACTCGGAATCATCGCT
